# Inferring transcriptional gene regulation network of starch metabolism in *Arabidopsis thaliana* leaves using graphical Gaussian model

**DOI:** 10.1186/1752-0509-6-100

**Published:** 2012-08-16

**Authors:** Papapit Ingkasuwan, Supatcharee Netrphan, Sukon Prasitwattanaseree, Morakot Tanticharoen, Sakarindr Bhumiratana, Asawin Meechai, Jeerayut Chaijaruwanich, Hideki Takahashi, Supapon Cheevadhanarak

**Affiliations:** 1 School of Bioresources and Technology, King Mongkut’s University of Technology Thonburi, Bangkok, 10140, Thailand; 2 National Center for Genetic Engineering and Biotechnology, Pathumthani, 12120, Thailand; 3 Department of Statistics, Faculty of Science, Chiang Mai University, Chiang Mai, 50200, Thailand; 4 Department of Chemical Engineering, Faculty of Engineering, King Mongkut’s University of Technology Thonburi, Bangkok, 10140, Thailand; 5 Department of Computer Science, Faculty of Science, Chiang Mai University, Chiang Mai, 50200, Thailand; 6 RIKEN Plant Science Center, Yokohama, 230-0045, Japan; 7 Department of Biochemistry & Molecular Biology, Michigan State University, 603 Wilson Rd, East Lansing, MI, 48824, USA

**Keywords:** *Arabidopsis thaliana*, Constans-like, Indeterminate domain 5, Graphical Gaussian model, Starch synthase 4, Transcriptional regulation

## Abstract

**Background:**

Starch serves as a temporal storage of carbohydrates in plant leaves during day/night cycles. To study transcriptional regulatory modules of this dynamic metabolic process, we conducted gene regulation network analysis based on small-sample inference of graphical Gaussian model (GGM).

**Results:**

Time-series significant analysis was applied for *Arabidopsis* leaf transcriptome data to obtain a set of genes that are highly regulated under a diurnal cycle. A total of 1,480 diurnally regulated genes included 21 starch metabolic enzymes, 6 clock-associated genes, and 106 transcription factors (TF). A starch-clock-TF gene regulation network comprising 117 nodes and 266 edges was constructed by GGM from these 133 significant genes that are potentially related to the diurnal control of starch metabolism. From this network, we found that β-amylase 3 (b-amy3: At4g17090), which participates in starch degradation in chloroplast, is the most frequently connected gene (a hub gene). The robustness of gene-to-gene regulatory network was further analyzed by TF binding site prediction and by evaluating global co-expression of TFs and target starch metabolic enzymes. As a result, two TFs, indeterminate domain 5 (AtIDD5: At2g02070) and constans-like (COL: At2g21320), were identified as positive regulators of starch synthase 4 (SS4: At4g18240). The inference model of AtIDD5-dependent positive regulation of *SS4* gene expression was experimentally supported by decreased *SS4* mRNA accumulation in *Atidd5* mutant plants during the light period of both short and long day conditions. COL was also shown to positively control *SS4* mRNA accumulation. Furthermore, the knockout of AtIDD5 and COL led to deformation of chloroplast and its contained starch granules. This deformity also affected the number of starch granules per chloroplast, which increased significantly in both knockout mutant lines.

**Conclusions:**

In this study, we utilized a systematic approach of microarray analysis to discover the transcriptional regulatory network of starch metabolism in *Arabidopsis* leaves. With this inference method, the starch regulatory network of *Arabidopsis* was found to be strongly associated with clock genes and TFs, of which AtIDD5 and COL were evidenced to control *SS4* gene expression and starch granule formation in chloroplasts.

## Background

Starch is an insoluble glucose polymer stored in seeds and storage organs of plants. The starch molecule is composed of two types of glucose polymers—amylose and amylopectin—and is organized to form a distinct structure called a starch granule. It is commonly accepted that biosynthesis of starch takes place during the day using an excess sugar residue from photosynthesis as a substrate. At night, starch granules in leaves are decomposed to sugars to be transported to seeds or storage organs and stored as reserved carbohydrates or used as precursors in other metabolic pathways [1-3]. Besides starch synthase, various enzymes and proteins have been identified to play unexpected roles in starch biosynthesis, metabolism and granule formation [4-11].

With regard to regulation of starch biosynthesis and metabolism, post-translational protein modifications have major impacts on controlling the enzyme activities [12-14]. Allosteric regulation of ADP-glucose pyrophosphorylase (AGPase) [15-17] and redox modulation of pullulanase-type debranching enzymes [18,19], glucan-water-dikinase (GWD) [20] and β-amylase [21] indicate the significance of post-translational regulatory mechanisms. Protein phosphorylation and formation of multi-protein complexes of starch synthase (SS), branching enzyme (BE), debranching enzyme (DBE), and starch phosphorylase (SP) suggest tight linkages of metabolic pathways through modification and physical interactions of the enzymes (reviewed in [12]). In addition to post-translational mechanisms, genes encoding starch metabolic enzymes are also known to be regulated under transcriptional control. In barley, a sugar-inducible transcription factor (TF) in the WRKY family, SUSIBA 2, is reported to act as an activator in endosperm starch biosynthesis [22]. In rice, a complex of a MYC protein (OsBP-5) and an EREBP protein (OsEBP-89) is proposed to be a transcriptional regulator of the rice *Wx* gene, whose product, namely the granule-bound starch synthase (GBSS), is responsible for synthesis of amylose in mature seeds [23]. Additional finding in *Arabidopsis* indicates that expression of the *GBSS-I* gene is controlled by 2 main clock TFs, circadian clock associated 1 (CCA1: At2g46830) and late elongated hypocotyl (LHY: At1g01060) [24]. The roles of these TFs suggest the significance of transcriptional mechanisms, although gene regulatory networks of starch metabolism remain largely uncharacterized.

Inference methods for construction of gene regulatory networks have been extensively developed after genome-wide microarray repositories became publicly available [25-31]. Reverse-engineering approaches of *Arabidopsis* gene regulatory network reconstruction utilizing large-scale microarray experiments have been previously proposed and revealed the *Arabidopsis* regulatory network models from different viewpoints [28-32]. Carrera and colleagues [30] applied a qualitative network model based on a probabilistic model and linear regression to 1,436 *Arabidopsis* microarrays, and analyzed topological parameters of the network. Their result showed that genes having cellular functions involved in responses and adaptation to environmental changes tended to have higher connectivity than genes not related to stress responses. Another approach of the gene network reconstruction from large-scaled *Arabidopsis* microarrays is proposed by Mao *et al.*[32]. They constructed a genome-wide co-expression network of *Arabidopsis* from 1,094 microarrays based on Pearson correlation and analyzed modular structures of the networks that are functionally related. Significantly enriched pathway terms were then analyzed for the predicted modules. One module was defined to be enriched in starch metabolism; 9 out of 10 genes contained in this module were related to starch metabolism. Since the genes in the same module were predicted from co-expression across various conditions, it is suggested that these starch metabolic genes are potentially co-regulated. The other method successfully utilized to construct a regulatory network is gene expression analysis using a modified graphical Gaussian model (GGM) [33,34]. The modified GGM is considered appropriate for analysis of microarray data that usually has a high-dimensionality problem (i.e. the number of genes is much higher than the number of measurements). This technique has been applied to 2,045 *Arabidopsis* microarrays to construct a gene network which can be subdivided to sub-network structures [29]. A number of sub-networks identified through this approach are suggested to be related to metabolism and stress responses. One of them is considered as a starch catabolism sub-network, where 7 out of 15 genes present in the network are apparently relevant to starch degradation pathways [29].

Given these backgrounds, systematic analysis of microarray data appears to provide insights into gene regulatory networks of starch metabolism in *Arabidopsis*. In this work, an inference model was constructed from a diurnal cycle microarray dataset to identify candidate transcriptional regulators of plant starch metabolism. This approach is based on evidence that biosynthesis and degradation of leaf starch is completed within 24 hours [3,35-37], and hypothesizes that regulators of our interests are co-expressed and oscillated with genes involved in starch metabolic processes under a day-night cycle [24,36-38]. Firstly, we identified genes whose expression profiles changed over an observed 24-hr time period. Among these temporally regulated ‘significant’ genes, a set of starch metabolic genes, TFs, and clock genes was utilized for the construction of gene association networks using the small sample inference framework of GGM [33,34]. Subsequently, a few pairs of TFs and starch metabolic genes were selected based on their correlation coefficients from global co-expression profiles. Finally, validation of the relationships between the selected TFs and starch metabolic genes were carried out using TF loss-of-function mutant lines. The results obtained from this study have led us to identify the involvement of TFs, indeterminate domain5 (AtIDD5: At2g02070) and constans-like (COL: At2g21320), in transcriptional regulation of an *Arabidopsis* starch metabolic gene. The work presented here provides a model for systematic understanding of regulatory networks of starch metabolic pathway applicable for modification of starch synthesis and accumulation.

## Results and Discussion

### Initial screening of significant genes in a diurnal cycle

A time-series significant analysis was performed using the Extraction of Differential Gene Expression (EDGE) software package. This software can identify differentially expressed genes from both typical and time-course microarray experiments [39,40]. In this research, the software was applied to detect changes in *Arabidopsis* gene expression occurring within a 24 hour period [36]. This data set is the time-course measurement of transcripts extracted from fully-expanded leaves of *Arabidopsis* grown under a 12-hour-light and 12-hour-dark (12 L/12D) condition. The samples are taken after 1, 2, 4, 8 and 12 hours in darkness or light, starting from the end or beginning of the light period, respectively. The concept of EDGE is based on the hypothesis testing of differential gene expression patterns fitted by natural cubic spline interpolation. Genes whose expression patterns deviate from a standard line within a 24-hour period would be detected as differentially expressed genes under a diurnal cycle. From approximately 22,000 *Arabidopsis* genes on the ATH-1 Affymetrix genome arrays, 1,480 genes were detected as significant genes (Q < 0.01). These significant genes were clustered by k-means clustering, then functionally classified in MapMan, a visualization tool for functional classification of *Arabidopsis*[41]. Genes encoding TFs and those related to starch metabolism were used as inputs for GGM network construction.

### Co-expression analysis of 1,480 significant genes using k-means clustering

**Figure 1:**
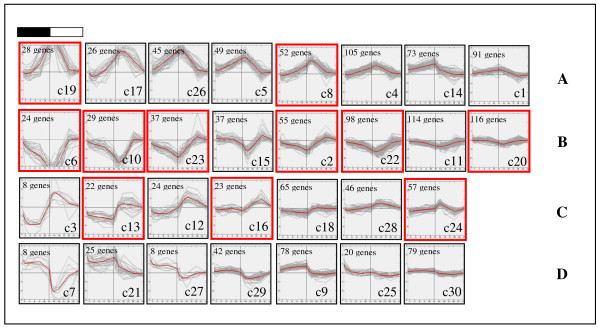
**Thirty clusters of 1,480 significant genes using k-means clustering (k = 30).** The number of gene members is shown at the top left of each cluster. The clusters where starch genes are present are indicated by red boxes. Four groups of 30 clusters were categorized by their expression patterns affected on light. (A) Tightly dark-positively and light-negatively regulated genes: the clusters of genes induced in the dark and repressed in the light periods. (B) Tightly dark-negatively and light-positively regulated genes: the clusters of genes repressed in the dark and induced in the light periods. (C) Light-positively regulated genes: the cluster of genes whose expressions are constant but the levels are elevated at dark to light phase transition. (D) Light-negatively regulated genes: the cluster of genes whose expressions are constant but the levels decline at dark to light phase transition. The black and white bar on the top of the figure corresponds to the dark and light period.

Since the leaf starch content of *Arabidopsis* increases in the light period and decreases in the dark period [36], we hypothesized that the high expression levels of genes in the starch biosynthetic pathway would be observed during the day, while those in the degradation pathway would occur at night. To test this hypothesis, the expression profile of all the significant genes were examined by k-means clustering (k = 30). All clusters were subjectively divided into 4 groups (Figure 1). Group A includes the genes whose mRNA levels are increased in the dark and decreased in the light. In contrast, the group B members showed their mRNAs increased in the light and decreased in the dark. Group C consists of the gene members whose expression remains relatively stable except their mRNA levels were observed to increase at the dark-to-light transition phase. Group D is similar to group C but the expression goes down at the dark-to-light transition phase. According to our hypothesis, the starch biosynthetic genes are expected to be clustered in group B and C since the gene expression increases when the light period starts. On the other hand, the genes in starch degradation whose expression are expected to increase in the dark period should be the members of group A and D.

According to Smith *et al.*[36], there are 48 genes related to starch metabolism. In the group of 1,480 significant genes identified in this study, 21 out of 48 starch metabolic genes were present, 9 of which are related to starch synthesis and 12 of which are known to function in starch degradation pathway (Table 1). By using k-means clustering, these starch metabolic genes were classified into 11 different clusters (Figure 1). Most of the starch synthesis genes (7 out of 9 genes) are observed in the clusters of groups B and C. This result supports the hypothesis that starch biosynthetic genes should be up-regulated during the day time. The two starch biosynthetic genes that do not follow this rule are those coding for granule-bound starch synthase (GBSS: At1g32900) and starch synthase 2 (SS2: At3g01180). The expressions of both genes show distinct diurnal patterns distinguishable from other starch synthase genes [36]. Since their products are reported to be embedded in the starch granules that are daily destroyed at the night period [36], their high expression levels after the onset of the light are considered necessary to regenerate GBSS and SS2 proteins for starch biosynthesis during the light period. In contrast, only 2 of 12 total genes encoding for enzymes in starch degradation pathways, α-amylase 2 (a-AMY2: At1g76130) and β-amylase 9 (b-AMY9: At5g18670), showed expression patterns correlated with the starch content profile. One explanation is that starch degrading enzymes might need a lag time in post-transcriptional or translational processes to become catalytically functional.

**Table 1:** Twenty-one differentially expressed starch genes (Q < 0.01) under the diurnal condition

**AGI**	**Name**	**Description**	**Cluster**	**Group**
At1g32900	GBSS	Granule-bound starch synthase	19	A
At1g76130	a-AMY2	α-amylase, putative	8	A
At3g01180	SS2	Starch synthase 2	8	A
At5g18670	b-AMY9	β-amylase, putative (BMY3)	19	A
At1g03310	ISA2/DBE1	Isoamylase, putative	2	B
At1g10760	GWD1	Glucan water dikinase	10	B
At1g69830	a-AMY3	α-amylase, putative	6	B
At2g36390	SBE2-1/BE3	1,4-α-glucan branching enzyme	22	B
At2g40840	DPE2	Glycoside hydrolase family 77 protein	23	B
At3g29320	StP, Plast	Glucan phosphorylase, putative	6	B
At3g46970	Stp, Cyt	Starch phosphorylase, putative	6	B
At4g09020	ISO3	Isoamylase, putative	6	B
At4g18240	SS4	Starch synthase 4	22	B
At5g11720	AGLU like-4	α-glucosidase1(AGLU1)	20	B
At5g24300	SS1	Starch synthase 1	2	B
At5g26570	GWD/ PWD	Glycoside hydrolase starch-binding domain-containing protein	10	B
At5g51820	PGM	Phosphoglucomutase	22	B
At5g64860	DPE1	disproportionating enzyme, putative	10	B
At2g32290	b-AMY6	β-amylase, putative	13	C
At2g39930	ISA1	Isoamylase, putative	16	C
At4g17090	b-AMY3	β-amylase (CT-BMY)	24	C

Cluster indicates the cluster number analyzed by k-means clustering (k = 30). All clusters were subjectively divided into 4 groups—A, B, C, and D—according to the expression pattern in each cluster (Figure 1).

### Functional categories of 1,480 significant genes

The 1,480 significant genes were categorized according to the functions defined in MapMan (Table 2). The largest functional group containing 471 genes, which accounted for ~32% of total significant genes, was classified as “Not assigned”. Out of the 471 genes in this group, 276 genes (~59%) were identified as unknown expressed proteins. The second largest group was the “RNA” group. This group contains 177 genes whose functions are related to RNA processing, transcription process, and regulation of transcription. As a result, 106 TF genes were assigned in the RNA group. Among 24 significant genes in the “Major carbohydrate metabolism” group (Table 2), 21 genes are starch-related, while the other 3 are related to sucrose metabolism. Additionally, our significant gene set also includes both clock and clock-regulated genes such as *circadian clock associated 1* (*CCA1*: At2g46830), *late elongated hypocotyl* (*LHY*: At1g01060), *early-flowering 3* (*ELF3*: At2g25930), *phytochrome B* (*PHYB*: At2g18790), *timing of cab expression 1* (*TOC1*: At5g61380), and *casein kinase II beta-chain* (*CKB3*: At3g60250). These TF, clock, and starch metabolic genes whose mRNA levels are significantly modulated during a diurnal cycle were used for reconstruction of transcriptional regulatory network of starch metabolism.

**Table 2:** Functional categories of 1,480 significant genes defined by the MapMan tool

**Functional category**	**Number of genes**	**Group**			
		**A**	**B**	**C**	**D**
Photosynthesis	33	9	8	12	4
Major carbohydrate metabolism	24	6	15	3	0
Minor carbohydrate metabolism	17	6	3	4	4
Glycolysis	10	0	9	1	0
Fermentation	2	0	2	0	0
Gluconeogenesis/glyoxylate cycle	3	1	0	0	2
Oxidative pentose phosphate pathway	4	0	3	0	1
TCA / organism Transformation	11	3	4	3	1
Mitochondrial electron transport /ATP synthesis	7	3	2	0	2
Cell wall	19	6	6	2	5
Lipid metabolism	41	13	12	9	7
N-metabolism	2	1	0	0	1
Amino acid metabolism	38	14	13	5	6
S-assimilation	2	2	0	0	0
Metal handling	7	3	3	1	0
Secondary metabolism	32	21	5	5	1
Hormone metabolism	36	11	13	8	4
Co-factor and vitamin metabolism	8	2	4	0	2
Tetrapyrrole synthesis	6	3	2	1	0
Stress	61	11	24	16	10
Redox regulation	26	9	11	3	3
Polyamine metabolism	6	4	1	1	0
Nucleotide metabolism	16	3	9	2	2
Biodegradation of xenobiotics	4	1	0	1	2
C1-metabolism	6	1	4	0	1
Miscellaneous	73	20	30	14	9
RNA	177	55	57	37	28
DNA	29	5	17	5	2
Protein	157	41	64	17	35
Signalling	74	25	24	12	13
Cell	53	9	26	5	13
Development	37	9	12	10	6
Transport	81	40	19	15	7
Not assigned	471	159	148	67	97

Groups A, B, C, and D correspond to classifications in k-means clustering analysis presented in Figure 1.

### Gene regulation network of starch metabolism in *Arabidopsis* leaves

To further investigate the transcriptional regulation of starch metabolic pathway, only metabolic genes in starch metabolism and all possible regulators (i.e. TF and clock genes) were focused in this research. The 133 significant genes —composed of 21 starch metabolic genes, 106 TF genes, and 6 clock genes— were subjected to GGM network construction [33]. Since GGM generates a conditional network depending on an input genes set, different sets of input genes are influent to the resulted network. Preservation of the TF-starch relationships in the networks constructed from expanded gene sets indicated the robustness of the network reconstructed by the focused set of starch metabolic and regulator genes (discussed in the following section).

**Figure 2:**
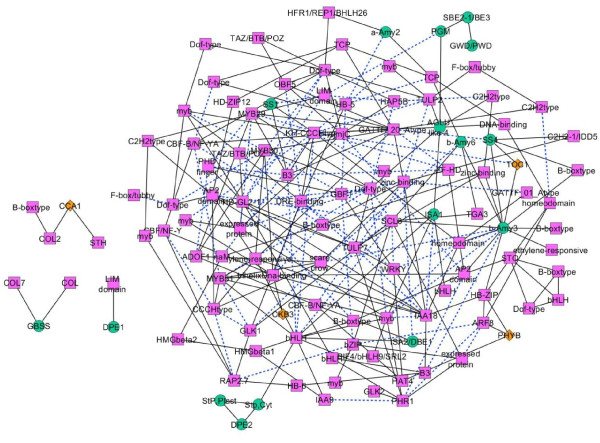
**The gene association network of starch metabolism inferred from GGM (Q < 0.05).** The network contains 117 nodes and 266 edges. A pink rectangle represents a TF gene; an orange diamond represents a clock gene; a green circle represent a starch gene; a black solid line represents an association with positive correlation; and a blue broken line represents an association with negative correlation.

The gene association network of starch metabolic pathway was constructed using small sample inference of GGM implemented in the R package ‘GeneNet’ [33]. The transcript profiles of 133 significant genes were retrieved from the original microarray data [36], and transformed to log-base 2 scales before inferring the gene association network. In the resulting network, a node represents a gene and an interaction between 2 genes is called an edge. Each edge indicates a correlated expression of any 2 genes after removing effects of other genes in a study set. The hypothesis testing of non-zero partial correlation and false discovery rate (FDR), multiple testing correction were employed to obtain significant correlation coefficients (i.e. significant edges) in the final network. From a total of 133 genes, the final network derived from GGM analysis at Q < 0.05 consisted of 117 nodes (or genes), corresponding with 16 starch (7 synthesis and 9 degradation), 97 TF, and 4 clock genes, and 266 edges (or, an association between 2 genes) (Figure 2). These edges could be the associations between 1) regulator and regulator genes (i.e. TF-TF, clock-TF, and clock-clock), 2) regulator and target genes (i.e. TF-starch and clock-starch), and 3) target and target genes (i.e. starch-starch). There are 215, 42, and 9 edges for the first, second, and third edge types, respectively.

Since 80% of the genes used in the network reconstruction are TF genes, predominant interactions in the final network represent those between TFs. To verify the significance of these TF-TF interactions and the robustness of the starch genes association network, especially the TF-starch relationships shown in Figure 2, another gene association network was reconstructed by expanding a set of metabolic genes from only genes in starch metabolism to genes in broader carbon-related metabolisms. The same set of 106 TFs, 6 clock genes, and 171 metabolic genes selected from 11 carbon-related functional groups categorized to be related to photosynthesis, major carbohydrate metabolism, minor carbohydrate metabolism, glycolysis, fermentation, gluconeogenesis/glyoxylate cycle, oxidative pentose phosphate pathway, TCA/organism transformation, mitochondrial electron transport/ATP synthesis, cell wall, and lipid metabolism by MapMan (Table 2) were utilized for network reconstruction. It should be noted that all 21 starch genes are present in the major carbohydrate metabolism category, thus included in the metabolic gene set. The gene association network of carbon-related metabolisms is shown in Additional file 1: Figure S1. In this expanded network, 151 out of 215 regulator- regulator edges (70%) and 26 out of 42 TF-starch or clock-starch relationships (62%) were identical with the starch network shown in Figure 2. All 6 TF candidates and their relationships to 5 starch genes that will be further experimentally validated (discussed in the following section) exist in the expanded network. The result indicates that most of the regulatory relationships of the starch gene association network are robust and preserved even when expanding an input gene set to carbon-related metabolic genes.

**Figure 3:**
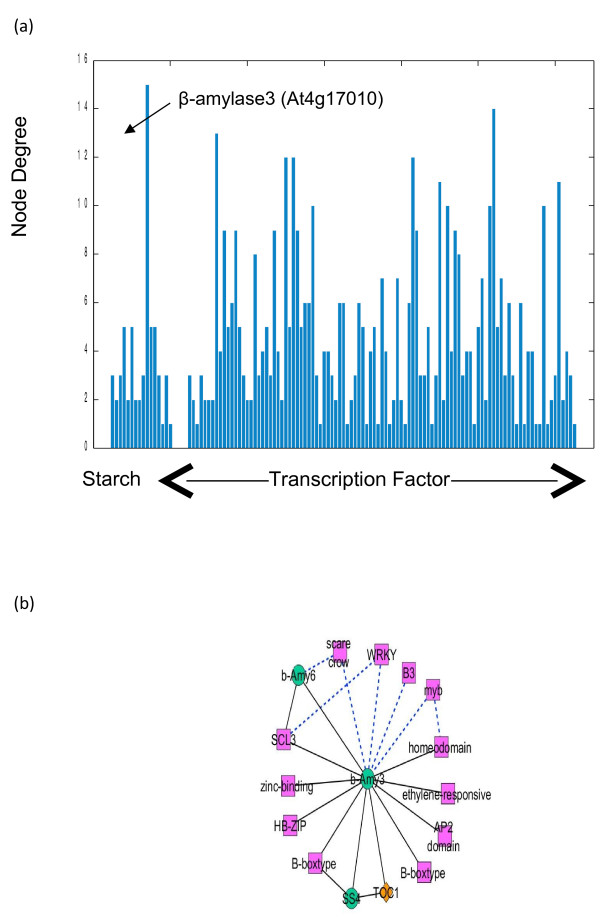
**β-amylase 3 is the most frequently connected gene (a hub gene). (a)** The node degree distribution of starch and TF genes in the gene association network. The gene with the highest degree of connection is β-amylase 3 (b-AMY3: At4g17090) as indicated by a black arrow. **(b)** Sub-network of the most-connected node (a hub gene), β-amylase 3 at Q < 0.05. A pink rectangle represents a TF gene; an orange diamond represents a clock gene; a green circle represents a starch gene; a black solid line represents an association with positive correlation; and a blue broken line represents an association with negative correlation.

According to Figure 3A, the node degree of TF and clock genes ranged from 1 to 14 connections. Among the starch metabolic genes, β-amylase 3 (*b-AMY3*: At4g17090) was detected as the most-connected node or a hub gene with 15 neighbors (Figure 3B). This result may indicate the importance of *b-AMY3* in the starch metabolism. The b-AMY3 represents a chloroplastic β-amylase [21,36,42] with a possible role in the leaf starch degradation process [3,43-45]. In the *b-AMY3* sub-network (Figure 3B), there are 2 starch metabolic genes, starch synthase 4 (*SS4*: At4g18240) and β-amylase 6 (*b-AMY6*: At2g32290), that showed positive correlations with *b-AMY3*. There are reports indicating that *SS4* involves in the starch granule initiation process [6,11], and *b-AMY6* is repressed under heat shock stress [46]. However, no functional correlation among these 3 starch metabolic genes has ever been reported.

In addition to gene-to-gene association with two starch metabolic genes, *b-AMY3* was also observed to have positive correlation with various TF genes and a clock gene, *TOC1*. *TOC1* is an evening gene expressed during the night period with a major role in circadian rhythm [47-49]. From the k-means clustering, expression of *b-AMY3* increased slightly during the night period, decreased at the dark-to-light transition phase, then increased again a few hours later. Our findings are not only in agreement with the results indicating regulation of *b-AMY3* expression under diurnal and circadian rhythms [36,50,51], but they also suggest the circadian control of *b-AMY3* via TOC1.

From the GGM network construction, we additionally found that the sub-networks of two starch metabolic genes encoding GBSS (At1g32900) and disproportionating enzyme (DPE1: At5g64860) and their TF neighbours are entirely separated from the rest of the network (Figure 2). These isolated modules indicate specific correlation between starch metabolic genes and their connected TFs. The LIM domain-containing protein (At2g39900) associated with DPE1 in the network is in fact WLIM2a, an actin-bundling protein that functions in cytoskeleton organization [52]; its gene expression is regulated by pickle (PKL), a member of CHD3 chromatin- remodelling protein involved in seed germination of *Arabidopsis*[53]. The other isolated module represents the association between *GBSS* and two zinc finger family proteins, constans-like (*COL*: At2g21320) and constans-like *7* (*COL7*: At1g73870). In rice, MYC and EREBP are known to act synergistically in transcriptional regulation of the rice *Wx* gene [23]. Since these homologues were not included in the *GBSS* sub-network of *Arabidopsis*, the results possibly suggest a difference in the mechanisms controlling the biosynthesis of storage starch (i.e. in rice endosperm) and transitory starch (i.e. in *Arabidopsis* leaves).

### Sub-networks for transcriptional regulation of starch metabolism

**Figure 4:**
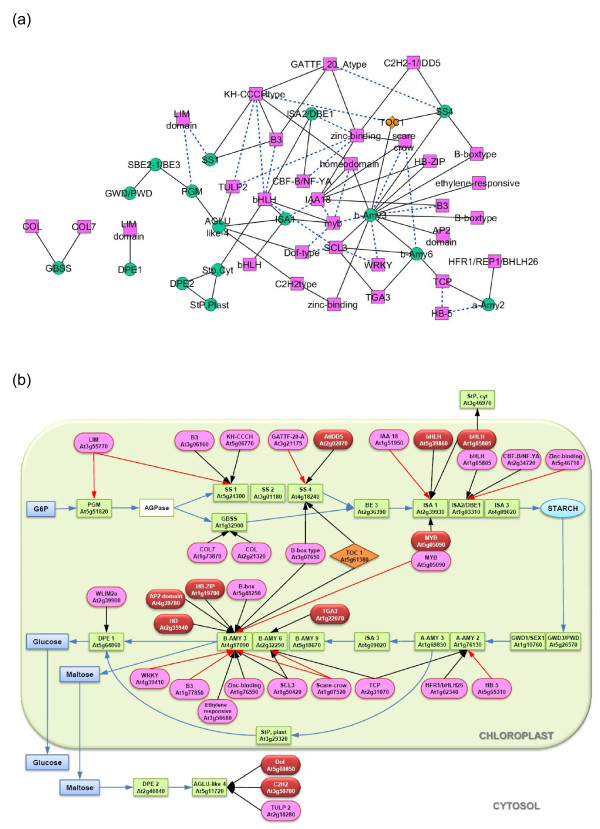
**The starch sub-network of 16 starch genes and their first step neighbours (Q < 0.05). (a)** The graphical display of the starch sub-network. A pink rectangle represents a TF gene; an orange diamond represents a clock gene; a green circle represents a starch gene; a black solid line represents an association with positive correlation; and a blue broken line represents an association with negative correlation. **(b)** The metabolic display of the starch sub-network. A pink oval represents a TF gene; A red oval represents a TF gene predicted to have physical binding sites in target genes according to the comparative TF family analysis; an orange diamond represents a clock gene; a green rectangle represents a starch gene; a blue rectangle represents a metabolite; a black line represents an association with positive correlation; a red line represents an association with negative correlation; and a blue line represents a reaction in starch metabolism.

To identify candidate TFs that may play regulatory roles in starch metabolism, we focused on the relationships between starch metabolic genes and their immediately connected TFs. The starch sub-network excerpted accordingly contained 49 nodes (16 starch, 1 clock, and 32 TF genes) and 79 edges (Figure 4A). Details of the genes in the starch sub-network are summarized in Additional file 2: Table S1. Within the starch sub-network, we observed 5 types of gene-to-gene interactions or edges; those were interactions between starch metabolic genes (8 edges), between TF genes (26 edges), between starch metabolic and TF genes (42 edges), between clock and starch metabolic genes (2 edges), and between clock and TF genes (1 edge).

The *Arabidopsis* gene networks were previously reconstructed from global microarray conditions based on GGM [29] and co-expression analysis [32]. Interestingly, the sub-networks related to starch metabolism were extracted from both studies, even though different algorithms were applied. The sub-networks derived from GGM [29] and co-expression analysis [32] contain a total of 15 and 10 genes, and from these gene set 10 and 9 were identified as starch metabolic genes, respectively. There are 6 genes in starch degradation, i.e. disproportionating enzyme 1 and 2 (At5g64860 and At2g40840, respectively), glucan water dikinase 1 (starch excess1 - At1g10760), glucan phosphatase (starch excess 4 - At3g52180), starch phosphorylase 2 (At3g46970), and α-amylase 3 (At1g69830), and one gene in starch synthesis, branching enzymes 3 (At2g36390) that both of these networks have in common. From total 15 starch metabolic genes identified in the sub-networks of previous studies, 7 genes, which are starch synthase 4 (At4g18240), branching enzyme 3 (At2g36390), disproportionating enzyme 2 (At2g40840), phosphoglucan water dikinase (At5g26570), isoamylase 2 (At1g03310), cytosolic and plastidial starch phosphorylase (At3g46970 and At3g29320, respectively), were also observed in our starch sub-network (Additional file 2: Table S1). These results suggest coherent expressions of starch metabolic genes, especially those in the starch degradation process. It is worth to note that the starch sub-networks derived from those studies contain mainly metabolic genes, except one clock-regulated gene encoding pseudo-response regulator 3 (APRR3: At5g60100) which was identified in the starch sub-network derived from the GGM analysis [29]. However, this clock gene was not present in our starch sub-network.

### Prediction of TF binding sites in promoter sequences of target genes

The 42 interactions between 12 starch and 32 TF genes were further analyzed by searching for the presence/absence of TF binding sites in the promoter region of starch metabolic genes (Table 3). The analysis conducted for prediction of physical binding of TF to target genes is TF family-based, thus, it stands on the assumption that different TFs of the same TF family most likely attach to the same TF-binding site on the promoter region of a target gene.

**Table 3:** The TF-starch relationships from the starch sub-network

**Starch Metabolic Gene (AGI code)**	**Starch Metabolic Gene (Gene Name)**	**pCor / Interaction**^**1**^	**TF (AGI code)**	**TF (Gene Name)**	**TF Family**	**AthaMap**^**2**^	**Rank of TF-starch correlation in ATTED**			
						**2-kb upstream**	**Correlation**	**Rank in all genes (total = 22,263 )**	**Ranks in all TFs (total = 1,849)**	**Rank in specific TF family/No. of TFs in a family**
At1g03310	ISA2/DBE1	-0.0756 / n	At5g46710	zinc-binding	PLATZ	NA	-0.05	11356	818	2/7
At1g03310	ISA2/DBE1	0.0674 / p	At2g34720	CBF-B/NF-YA	CCAAT-HAP2	NA	0.03	8424	557	6/10
At1g03310	ISA2/DBE1	0.0733 / p	At1g05805	bHLH	bHLH	N	0.165	4598	241	27/114
At1g32900	GBSS	0.0736 / p	At1g73870	COL7	C2C2-CO-like	NA	0.609	17	4	3/42
At1g32900	GBSS	0.0686 / p	At2g21320	COL	C2C2-CO-like	NA	0.608	18	5	4/42
At1g76130	a-AMY2	0.0665 / p	At2g31070	TCP	TCP	N	0.174	3347	344	7/20
At1g76130	a-AMY2	0.0758 / p	At1g02340	HFR1/BHLH26	bHLH	N	-0.019	11812	1052	56/114
At1g76130	a-AMY2	-0.1268 / n	At5g65310	HB-5	HB	N	-0.372	21286	1809	87/89
At2g32290	b-AMY6	0.0763 / p	At1g22070	TGA3	bZIP	Y	0.111	3654	225	4/69
At2g32290	b-AMY6	0.0676 / p	At1g50420	SCL3	GRAS	NA	0.07	4806	328	10/32
At2g32290	b-AMY6	-0.0707 / n	At1g07520	scarecrow	GRAS	NA	-0.125	18495	1527	30/32
At2g32290	b-AMY6	0.0818 / p	At2g31070	TCP	TCP	N	0.204	2008	111	4/20
At2g39930	ISA1	0.0917 / p	At5g05090	myb	GARP-G2-like	Y	0.242	3728	181	6/39
At2g39930	ISA1	0.0763 / p	At1g05805	bHLH	bHLH	Y	0.356	2106	81	9/114
At2g39930	ISA1	0.0783 / p	At5g39860	bHLH	bHLH	Y	0.255	3490	164	22/114
At2g39930	ISA1	-0.0704 / n	At1g51950	IAA18	AUX-IAA	NA	0.056	8010	542	14/28
At3g46970	Stp,Cyt	0.0670 / p	At1g05805	bHLH	bHLH	Y	0.442	511	37	1/114
At4g17090	b-AMY3	0.0761 / p	At2g35940	Homeo domain	HB	Y	0.233	2173	127	12/89
At4g17090	b-AMY3	0.0674 / p	At1g19700	HB-ZIP	HB	Y	0.009	7815	644	41/89
At4g17090	b-AMY3	0.0916 / p	At4g39780	AP2 domain	AP2-EREBP	Y	-0.179	18803	1537	110/130
At4g17090	b-AMY3	0.0810 / p	At3g58680	ethylene-responsive	MBF1	NA	-0.009	8743	713	1/3
At4g17090	b-AMY3	0.1093 / p	At1g76590	zinc-binding	PLATZ	NA	0.015	7522	608	2/7
At4g17090	b-AMY3	-0.0871 / n	At1g77850	B3	ARF	NA	0.026	7013	569	7/18
At4g17090	b-AMY3	0.0684 / p	At5g48250	B-box	C2C2-CO-like	NA	0.285	1615	74	9/42
At4g17090	b-AMY3	0.0671 / p	At3g07650	B-box	C2C2-CO-like	NA	0.264	1805	92	10/42
At4g17090	b-AMY3	0.1051 / p	At5g61380	TOC1	C2C2-CO-like	NA	0.181	2903	191	14/42
At4g17090	b-AMY3	-0.0941 / n	At5g05090	myb	GARP-G2	NA	0.015	7574	611	19/39
At4g17090	b-AMY3	-0.0774 / n	At1g07520	scarecrow	GRAS	NA	-0.139	16919	1385	30/32
At4g17090	b-AMY3	-0.0757 / n	At4g39410	WRKY	WRKY	N	0.094	4649	365	16/63
At4g18240	SS4	0.0928 / p	At2g02070	AtIDD5	C2H2	Y	0.542	158	9	1/122
At4g18240	SS4	-0.0671 / n	At3g21175	GATTF_20_A	ZIM	NA	0.225	2276	119	1/16
At4g18240	SS4	0.1016 / p	At5g61380	TOC1	C2C2-CO-like	NA	0.569	124	5	3/42
At4g18240	SS4	0.0720 / p	At3g07650	B-box	C2C2-CO-like	NA	0.514	201	13	5/42
At5g11720	AGLU-like4	0.0687 / p	At3g50700	C2H2	C2H2	Y	0.295	2024	87	5/122
At5g11720	AGLU-like4	0.0837 / p	At5g60850	Dof	C2C2-Dof	Y	0.135	5244	310	6/32
At5g11720	AGLU-like4	0.0910 / p	At2g18280	TULP2	TLP	NA	0.213	3463	169	3/11
At5g24300	SS1	0.0961 / p	At5g06770	KH-CCCH	C3H	NA	0.422	1194	31	3/172
At5g24300	SS1	-0.0786 / n	At3g55770	LIM	LIM	NA	-0.079	13020	995	7/13
At5g24300	SS1	0.0817 / p	At3g06160	B3	ABI3-VP1	N	0.241	2965	136	4/45
At5g51820	PGM	-0.0829 / n	At3g55770	LIM	LIM	NA	-0.07	11712	853	6/13
At5g64860	DPE1	0.0663 / p	At2g39900	WLIM2a	LIM	NA	0.715	30	1	1/13

Global-expression correlations extracted from ATTED database and the correlation ranks were shown in the table.

1. pCor is partial correlation of a gene pair under the diurnal condition predicted by GGM. p and n represent a positive and negative interactions, respectively.

2. Y = Physical binding of TFs to target genes was predicted by the comparative TF family analysis; N = No physical binding of TFs was predicted; NA = no TF binding site information in AthaMap.

In the TF family-based comparative analysis, all possible plant TF binding sites in the 2-kb upstream region of the 12 target genes (i.e. starch metabolic genes) were first obtained using a web-based tool from AthaMap database http://www.athamap.de/[54-57]. The names of known TFs and their relative binding locations predicted within the 2-kb upstream region of 12 starch metabolic genes are summarized in Additional file 3: Table S2. From the list of all possible TF binding sites, physical binding was predicted between 10 TFs and 6 starch metabolic genes, shown as 11 starch-TF interactions in the starch sub-network (Table 3). For easier visualization, we included information on the presence of putative TF-binding sites and re-drew the regulatory model of genes in the starch sub-network (Figure 4B). The results indicate that 10 TFs show only a positive correlation with 6 starch metabolic genes and can be classified into 7 families.

### Predicted regulatory modules for a model validation

The robustness of the predicted regulatory network model of starch metabolism was further verified using a diverse set of “condition-independent” microarray data. This analysis has been implemented to find how the expression patterns of any 2 genes under various conditions correlate. The data representing the relationship of *Arabidopsis* co-regulated genes was obtained from the ATTED-II database http://atted.jp/[58,59]. In ATTED-II, the pair-wise correlation coefficients of 22,263 *Arabidopsis* genes were calculated from 58 experiments of GeneChip microarray (1,388 arrays in total) using weighted Pearson correlation. The pair-wise correlation coefficients of 12 starch metabolic genes with all *Arabidopsis* TF genes (1,849 genes listed in this database) were ranked from highest to lowest value (Table 3).

The pair-wise correlation coefficients between given target genes and all TFs were observed as normally distributed in this condition-independent analysis. The TF was thus considered significant when its correlation coefficient with its target starch metabolic gene was higher than the population mean with 97.5% confidence analysed by the single-sample *t*-test (one-tailed). From all the TFs in the starch sub-network, 8 TF genes passed this cut-off by being significantly and highly correlated with their target gene expression, and they were preliminarily chosen as candidates for experimental validation (discussed in the following section). However, among these 8 candidates, the expression patterns of 2 TF genes, constans-like 9 (*COL9*: At3g07650) and *bHLH* (At1g05805), did not adhere to the simple assumption that a TF should express at the same time, or earlier, than its target gene. The diurnal expression patterns of both *COL9* and *bHLH* indicated that their induction took place after the expression of their potential target starch metabolic genes (Additional file 4: Figure S2). Therefore, by excluding these 2 TFs, the rest 6 candidate TF genes—*AtIDD5* (At2g02070), *C2H2* (At3g50700), *COL* (At2g21320), *COL7* (At1g73870), *WLIM2a* (At2g39900), and *KH-CCCH* (At5g06770), each predicted as regulators for the expression of *SS4*, α-glucosidase-like 4 (*AGLU-like 4*: At5g11720), *GBSS* (co-regulated by *COL* and *COL7*), *DPE1*, and starch synthase 1 (*SS1*: At5g24300), respectively (Table 4)—were selected as final candidates and subsequently used in experimental validation. According to the TF family-based prediction of binding sequences (Tables 3 and 4) of these 6 candidates, putative binding sites for zinc finger C2H2 type TFs were located in the promoter regions of their target genes, *SS4* and *AGLU-like 4*. Accordingly, the gene-to-gene associations between *AtIDD5* and *SS4*, and between *C2H2* and *AGLU-like 4*, gain strong support from both the TF binding site prediction and the global co-expression analysis.

**Table 4:** Six candidate TF genes and their ranks based on the global-expression correlations

**Starch metabolic gene**		**TF gene**		**TF family**	**Binding Site**^**1**^	**ATTED Correlation**^**2**^	**TF rank**^**3**^	**Family rank**^**4**^
At4g18240	SS4	At2g02070	AtIDD5	C2H2	Y	0.542	9	1/122
At5g11720	AGLU- like 4	At3g50700	C2H2	C2H2	Y	0.295	87	5/122
At5g64860	DPE 1	At2g39900	WLIM2a	LIM	n.a.	0.715	1	1/13
At1g32900	GBSS	At1g73870	COL7	C2C2-CO-like	n.a.	0.609	4	3/42
At1g32900	GBSS	At2g21320	COL	C2C2-CO-like	n.a.	0.608	5	4/42
At5g24300	SS1	At5g06770	KH-CCCH	C3H	n.a.	0.422	31	3/172

1. Y = potential binding site of a TF was found at the 2,000-base upstream region of target gene by the comparative TF family analysis; n.a. = information of a binding site of that TF is not available in AthaMap http://www.athamap.de/.

2. Pearson correlation calculated from the 58 GeneChip experiments including 1,388 arrays using the weighted Pearson correlation in ATTED database http://atted.jp/.

3. Rank of TF based on the ATTED correlation of 1,849 TF genes in the database.

4. Rank of TF based on the ATTED correlation of TF genes in each specific TF family. The numerator indicates the rank and the denominator indicates the number of genes in a specific TF family.

### Expression analysis of target genes predicted in the regulatory modules

To experimentally verify the regulatory role of the 6 candidate TFs in the proposed TF-target model (Figure 4B; Table 4), the accumulation of starch metabolic gene transcripts was determined using homozygous knockout lines. T-DNA or transposon inserted knockout lines—*Atidd5*, *c2h2*, *col*, *col7, wlim2a*, and *kh-ccch*—were obtained from The *Arabidopsis* Biological Resource Centre (ABRC) [60] and The Nottingham Arabidopsis Stock Centre (NASC) [61]. The homozygous mutant plants were grown under the conditions described in [36] (See method). Rosette leaves at 3.90 developmental stage [62] were harvested 4 times within a 24- hour period, and used as materials for RNA extraction. The mRNA levels of TFs and target genes were quantified by quantitative real-time reverse transcription polymerase chain reaction (qRT-PCR) analysis.

**Figure 5:**
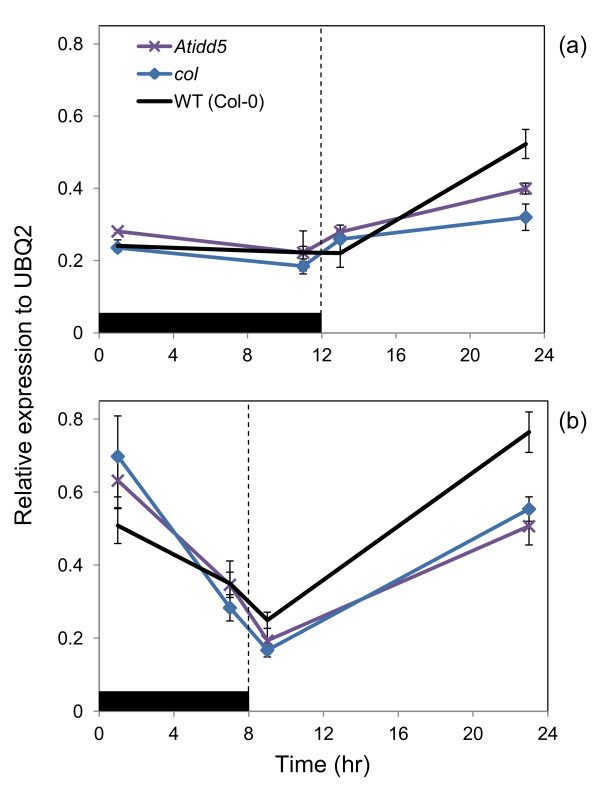
***SS4* gene expression in the wild type, *Atidd5* and *col *mutants.** The relative transcript level of SS4 mRNA was measured in *Arabidopsis* grown under **(a)** short (12 L/12D) and **(b)** long day (16 L/8D) conditions. Ubiquitin 2 (UBQ2) was used as a constitutive control for normalization.

*AtIDD5*, *C2H2*, *COL7*, and *KH-CCCH* mRNAs were absent in *Atidd5*, *c2h2*, *col7*, and *kh-ccch* mutant lines, respectively, whereas *COL* and *WLIM2a* mRNAs were partially detected in *col* and *wlim2a* mutant lines, respectively (data not shown). The mRNA accumulations of target starch metabolic genes were then monitored in these mutant lines to determine the effect of disruption of TFs predicted to act as regulators. The results indicated that a starch metabolic gene, *SS4*, showed a decreased mRNA level in *Atidd5* mutant (Figure 5A). Significant down-regulation of *SS4* was observed at the end of the light period of both short and long days (Figures 5A and 5B). It appears that *AtIDD5* plays an important role in the regulation of *SS4* gene expression. By contrast, the mRNA levels of *AGLU-like 4*, *GBSS*, *DPE1*, and *SS1* genes were not different between the wild type and mutant lines—*c2h2*, *col* &*col7*, *wlim2a*, and *kh-ccch*, respectively—during the time course of 12 L/12D condition (Additional file 5: Figure S3).

In addition to the effect of direct TF-target relationships, the regulatory network can be affected by the associated modules particularly when the target genes are in the same metabolic process. Based on this assumption, the *SS4* mRNA levels were determined in other TF mutant lines. Similar to the results obtained from the *Atidd5* mutant, *SS4* was down-regulated in the *col* mutant during the light period of both short and long day conditions (Figure 5A and 5B). Alteration of *SS4* expression may have resulted from (i) the negative effect of disruption of COL on *AtIDD5* gene expression or (ii) the direct control of *SS4* by COL. Since the mRNA levels of *AtIDD5* in the wild type and the *col* mutant were different only in the long day condition (Additional file 6: Figure S4), regulation of *AtIDD5* expression by COL therefore remains inconclusive. Although the exact underlying mechanism is unknown, COL appears to be in part of the starch metabolic gene regulatory network, and its relevance is further evidenced by starch granule deformation (discussed in the following section).

According to *in silico* prediction of the *Arabidopsis* proteome, 176 proteins are classified in the C2H2 zinc finger family [63]. *AtIDD5* belongs to this C2H2 gene family and is further classified into the same sub-family as the maize indeterminate 1 gene (*ZmID1*), which is a key regulator of flowering transition [63,64]. In *Arabidopsis*, there are 16 homologues of *AtIDD* genes. Among them, the biological functions of magpie (*MGP*/*AtIDD3*; At1g03840), nutcracker (*NUC*/*AtIDD8*; At5g44160), and jackdaw (*JKD*/*AtIDD10*; At5g03150) genes have been characterized [65,66]. To date, there are only three *AtIDD* genes—*NUC/AtIDD8*, *AtIDD14* (At1g68130), and shoot graviropism 5 (*SGR5*/*AtIDD15*; At2g01940)—that have been reported to play roles, though indirectly, in sugar and starch metabolism [67-69]. Based on phylogenetic analysis, the *AtIDD* genes most closely related to *AtIDD5* are *AtIDD4* (At2g02080) and *AtIDD6* (At1g14580). AtIDD4 is reported as a TF whose expression is affected by defects in chloroplast import machinery, and it is postulated to function as a transcriptional activator of nuclear-encoded photosynthetic gene expression [70]. In addition, *AtIDD4* and *AtIDD6* are identified as gibberellin-regulated genes [71]. Based on the evidence known to date, both AtIDD4 and AtIDD6 do not seem to have any function related to sugar and starch metabolism.

The database of global gene expression analysis provides evidence showing that *AtIDD5* is abundantly expressed in leaf tissues. The global view of *AtIDD5* gene expression was roughly examined using the Genevestigator web-based software [72]. The expression of *AtIDD5* was observed ubiquitously in all stages, but its level was particularly high from stages of developed rosette leaves to developed flowers. In flowers, expression pattern of *AtIDD5* was classified as ‘stamen-specific lack of expression’, suggesting that its expression disappears, especially in anthers of flowers from stage 7 to 11 [73,74].

Information on AtIDD5-interacting proteins further suggests that AtIDD5 is associated with other signalling components, such as radical-induced cell death 1 (RCD1: At1g32230) [75]. RCD1 is not only known as clone eighty-one (CEO1), which recovers the oxidative stress-sensitivity phenotype of the Yap1^-^ mutant yeast [76], but also a major regulator of hormonal signalling and stress-response processes [77,78]. According to the AGRIS database http://arabidopsis.med.ohio-state.edu/REIN/, AtIDD5 is predicted to interact with a MADS-box domain TF, sepallata 3 (SEP3), which is a global moderator of multifunctional protein-complexes controlling flowering and hormonal signaling processes, especially responses to auxin stimuli [79].

### Total starch content and granule morphology analysis

**Figure 6:**
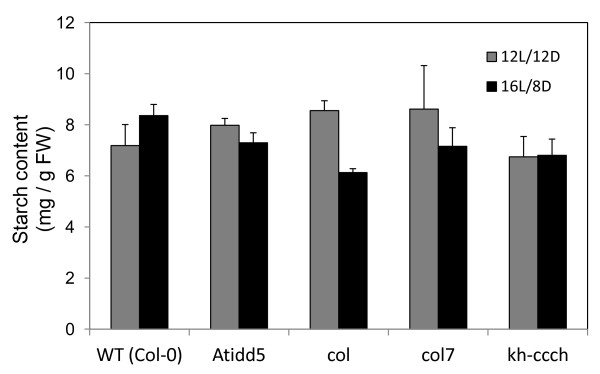
**Total starch content of leaves in the wild type and *Atidd5*, *col*, * col7*, and *kh-ccch* mutants.***Arabidopsis* plants were grown under short (12 L/12D; grey bar) and long day (16 L/8D; black bar) conditions, respectively.

Total starch content was measured under both short and long day conditions. Starch was extracted from fully expanded leaves of all the mutants and the wild type, and analyzed in the form of glucose by capillary electrophoresis-diode array detector (CE-DAD) after enzymatic digestion (see method). Both the mutants and the wild type accumulated starch at relatively similar levels (Figure 6), even though *SS4* was down-regulated in *Atidd5* and *col* mutants (Figure 5). It has been reported that the size of starch granules can be significantly altered in *ss4* mutant while only 35% reduction of the starch content could be observed [6]. Referring to these previous findings, we speculated that changes in starch granule morphology and number may occur in *Atidd5* and *col* mutants as they show reduced levels of *SS4* mRNA accumulation during the light period (Figure 5).

**Figure 7:**
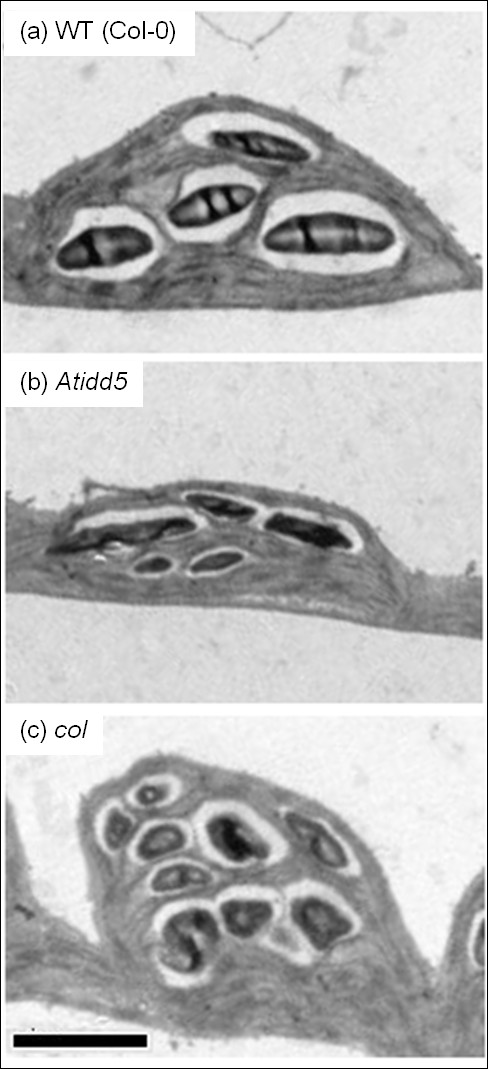
**Transmission electron micrographs of the wild type, *Atidd5*, and *col *chloroplast.** Length of a scale bar indicated in the micrograph is 2 μm.

Chloroplast and starch granule morphology of *Atidd5* and *col* mutant lines were examined by transmitted electron microscopy (TEM). Transmission electron micrographs of *Atidd5* and *col* mutant lines and wild type are shown in Figure 7. They were analyzed by the image processing software Image J (version 1.45) to obtain a group of data sets including (i) size measured by ‘Area’ and (ii) shape measured by ‘Width’, ‘Height’, and ‘Circularity’ of the chloroplast and starch granule cross-sections. Although these parameters might not represent the actual size and shape of chloroplasts and starch granules, we considered them suitable for a comparative purpose. Since the data was not normally distributed, a non-parametric statistic, the Mann–Whitney *U* test, was applied for testing significant differences between the wild type and the mutants. The relative mean ranks and P-values from the Mann–Whitney *U* test are described in Table 5. Descriptive statistics of chloroplast and starch granule morphology of *Atidd5* and *col* mutants are summarized in Additional file 7: Table S3.

**Table 5:** **The statistics from Mann–Whitney*U*test of chloroplast and starch morphology, and granule number**

	***Arabidopsis* Line**	**N**^**1**^	**Area (μm**^**2**^**)**	**Width (μm)**	**Height (μm)**	**Circularity**
CHLOROPLAST			Relative Mean Rank^2^			
	*Atidd5*	259	35.52	41.96	45.06	42.75
	WT (Col-0)	368	60.33	55.80	53.61	55.24
	*col*	176	38.81	53.70	33.94	48.20
	WT (Col-0)		55.49	48.37	57.82	50.99
			P-value (Mann–Whitney U)^3^			
	*Atidd5*		**3.38E-26**	**3.52E-09**	**2.60E-04**	**9.86E-08**
	*col*		**2.99E-10**	0.0442	**1.90E-19**	0.2919
STARCH GRANULE			Relative Mean Rank			
	*Atidd5*	890	45.63	47.58	47.35	47.66
	WT (Col-0)	895	54.40	52.47	52.69	52.38
	*col*	505	48.82	54.72	38.91	60.53
	WT (Col-0)		50.72	47.40	56.31	44.11
			P-value (Mann–Whitney U)			
	*Atidd5*		**1.38E-10**	**3.45E-04**	**9.54E-05**	**5.54E-04**
	*col*		0.2352	**5.23E-06**	**2.49E-27**	**1.64E-24**
GRANULE NUMBER			Number of starch granule / chloroplast			
			Relative Mean Rank			
	*Atidd5*	283	53.57			
	WT (Col-0)	408	47.65			
	*col*	177	54.18			
	WT (Col-0)		48.31			
			P-value (Mann–Whitney U)			
	*Atidd5*		**0.0067**			
	*col*		**0.0213**			

1. N is a number of observed chloroplast (or starch granule) from Transmission electron micrographs. For the granule number analysis, N is a number of observed chloroplast.

2. Relative Mean Rank was calculated from a ratio of mean rank to total rank and multiplied by 100 when the data was ranked in an ascending order.

3. The P-value calculated from the Mann–Whitney *U* test less than 0.05 was shown in bold letters indicating a significant difference of morphological parameters between the mutant and the wild type.

Area, width, height, and circularity of 259, 176, and 368 chloroplasts of *Atidd5*, *col*, and the wild type, respectively, were measured using the ImageJ software. According to the Mann–Whitney U analysis, chloroplast area of the wild type was significantly larger than that of *Atidd5* and *col* mutants (P-value = 3.38E-26 and 2.99E-10, respectively) (Table 5) with the means of chloroplast areas at 13.18, 9.26, and 10.50 μm^2^ for the wild type, *Atidd5*, and *col*, respectively. In addition to the size, the shape of chloroplasts of both mutants also differed from the wild type. The width, height, and circularity of the chloroplasts were significantly smaller in *Atidd5* than in the wild type. The small circularity values of *Atidd5* chloroplasts indicate that they are in more oblong shapes relative to the wild type chloroplasts. In addition, the chloroplasts in the *col* mutant had longer width but less height than those in the wild type. The results, therefore, suggest that both mutants develop chloroplasts with altered morphology, which, particularly, appear smaller or thinner than the chloroplasts in the wild type (Figure 7 and Table 5).

Since chloroplasts of both mutants were altered with respect to their size and shape, we examined their effects on the morphology of accumulated starch granules. Reduction of starch granule size, inferred from the cross-section area, was significant in *Atidd5* (P-value = 1.38E-10), but not in the *col* mutant. The means of starch granule areas of the wild type, *Atidd5*, and *col* were 0.54, 0.42, and 0.50 μm^2^, respectively. In contrast, the granule shape deformity was noticed in both *col* and *Atidd5* mutants (Figure 7 and Table 5). The decrease in width, height, and circularity of *Atidd5* starch granule most likely suggested that the granule was small and in oblong shapes. As compared to the wild type, the *col* starch granules were observed to have greater circularity, suggesting that they were relatively round in shape.

**Figure 8:**
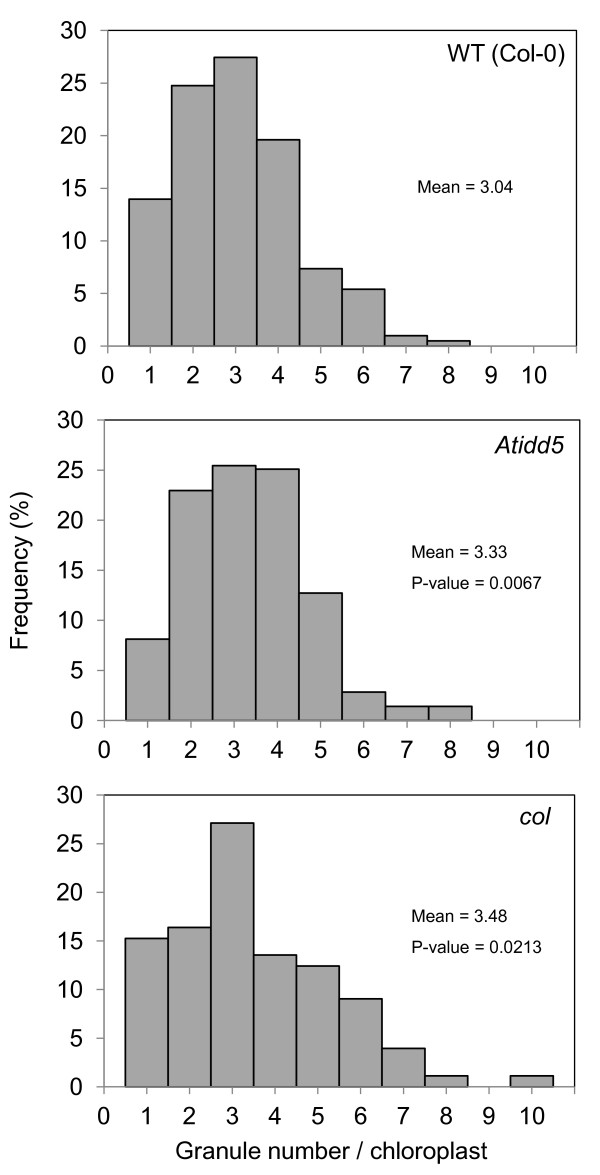
**Distribution of granule number per chloroplast in the wild type, *Atidd5*, and* col* mutants.** Means and P-value calculated from the Mann–Whitney *U* test are indicated.

According to the work of Rolden and coworkers [6], a chloroplast of the *ss4* mutant mostly contains one large starch granule. When examined under TEM—among 283, 177, and 408 chloroplasts of *Atidd5*, *col*, and the wild type, respectively—none of the chloroplasts were observed to contain a single large starch granule like the *ss4* mutant. On the other hand, the majority of chloroplasts—corresponding to 25.4%, 27.1% and 27.5% of observed chloroplasts in *Atidd5*, *col,* and the wild type, respectively—normally contained 3 starch granules. We further investigated the distribution of starch granule number per chloroplast to find the difference between the mutants and wild type (Figure 8). In the *Atidd5* mutant, 86.2% of the observed chloroplasts contained 2–5 starch granules, whereas 85.8% of chloroplasts from the wild type contained 1–4 starch granules. Interestingly, we observed that the number of chloroplasts containing 2 and 4 starch granules in the *col* mutant was lower than those in the *Atidd5* and the wild type, whereas the number of chloroplasts containing more than 5 granules was higher than the other lines (Figure 8). Moreover, the *col* mutant was the only line that was observed to contain up to 10 starch granules per chloroplast. The relative mean rank and P-value from the Mann–Whitney *U* test of the mutants and the wild type shown in Table 5 indicated that both *Atidd5* and *col* mutants had significantly higher numbers of starch granules per chloroplast than the wild type (P-value = 0.0067 and 0.0213, respectively). The results suggest that reduction of *SS4* expression in the *Atidd5* and *col* mutant lines leads to a significant increase in starch granule numbers, while their distributions of granule number per chloroplast are differently affected among the mutants (Figure 8).

**Figure 9:**
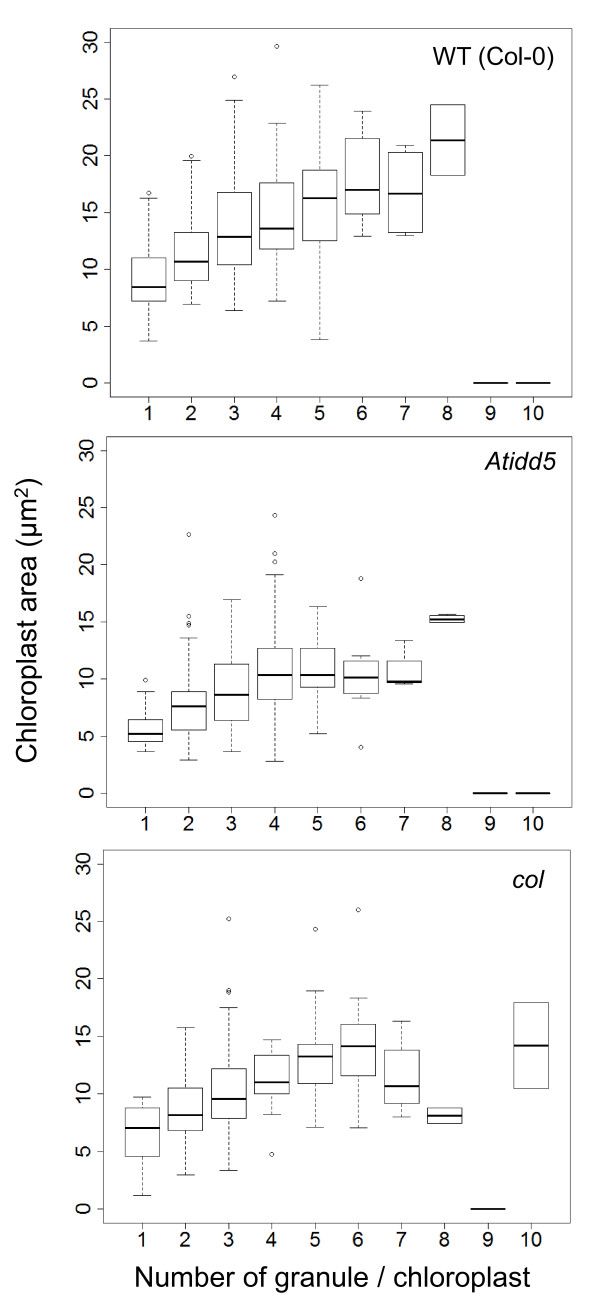
**Relationship between granule number and chloroplast size in the wild type, * Atidd5*, and* col* mutants.**

The relationship between the size of chloroplast and the number of accumulated starch granules is shown in Figure 9. Our results indicate that the number of starch granules increased according to chloroplast size. Larger chloroplasts tended to contain greater numbers of starch granules; however the pattern of correlation was not uniform among the wild type and mutants. In the *Atidd5* mutant, a positive correlation between chloroplast size and the number of starch granules was only observed in the chloroplast containing 1–4 granules. It appears that the chloroplasts in this mutant are unable to expand after reaching critical size; however, they continued to store higher numbers of starch granules without increasing their size. In *col* mutant, the positive correlation was observed in the chloroplast containing 1–6 granules, whereas the size of the chloroplast containing 6–8 starch granules tended to decrease in accordance with an increase in the number of starch granules. The average sizes of chloroplasts containing 7 and 8 granules were the same as those having 2 starch granules. In addition, the size of the *col* chloroplasts containing 10 starch granules was similar to the size of chloroplasts containing 6 granules, suggesting this might be the critical size limit of the *col* chloroplast.

The results indicated that, in addition to having defects in their control of *SS4* gene expression, both *Atidd5* and *col* mutants are unable to increase the size of chloroplasts, although they may still retain the capability to expand their chloroplast to contain relatively small numbers of starch granules until the chloroplast reaches its critical size limit. Particularly in *Atidd5*, having relatively small starch granules can be another adaptive response caused by chloroplast deformity. The observed phenomena may suggest the alternative roles of AtIDD5 and COL in controlling chloroplast size limit, which may synchronize with transcriptional regulation of a starch biosynthetic enzyme, SS4. Our findings address a question of how starch biosynthesis and chloroplast development and/or functions are synergistically controlled in plant cells. The underlying mechanism of interaction awaits further investigation.

## Conclusions

In this study, we proposed a transcriptional regulatory network of starch metabolism in *Arabidopsis* leaves, and examined the biological relevance of predicted network modules. The general workflow of data acquisition, refinement, and experimental validation provides a model case for reconstruction of transcriptional regulatory network. The present work widely utilizes publicly available biological information and resource databases, demonstrating how they can be integrated to find biological significance of predicted network modules. Construction of gene-to-gene association network models is based on diurnal regulation of starch metabolism in leaves where the transcriptomes oscillate during the day/night cycles. We first grouped time-series-dependent significant genes on transcriptome into four classes showing distinct patterns of co-regulation with starch biosynthesis or degradation. A particular focus has been placed on relationships between TFs, clock genes and starch metabolic genes, to obtain transcriptional regulatory network model of starch metabolism. The network constructed by the small sample inference of GGM suggests relationships between TFs and target starch metabolic genes. Gene-to-gene associations have been further refined by prediction of TF binding sites in target genes and by global co-expression analysis. Through these approaches, we finally showed the involvement of AtIDD5 and COL in transcriptional regulation of *SS4*. These regulatory networks were considered attributable to daytime starch biosynthesis by SS4. In addition, AtIDD5 and COL were shown to control chloroplast development and starch granule formation. The present work on TF network modelling and examination provides new insights into the regulatory mechanisms of starch biosynthesis and granule formation in the chloroplast.

## Methods

### Microarray data pre-processing

This study utilized *Arabidopsis* Affymetrix microarray data (CEL files) downloaded from the Nottingham *Arabidopsis* Stock Centre's microarray database (NASCArrays) [Experiment Reference Number: NASCARRAYS-60] http://affymetrix.arabidopsis.info/. This microarray experiment contains a set of 22 k *Arabidopsis* ATH-1 genome array transcriptome data of leaves at developmental stage 3.9 taken after 1, 2, 4, 8, and 12 hours in both darkness and light [36]. A ‘qspline’ normalization [80] and model-based expression index [81] were carried out in the microarray pre-processing, which was done using the Affy package in Bioconductor http://www.bioconductor.org.

### Significant analysis of time-series data

The significant test for the time-series data was performed using the EDGE program (version 1.1.175) [39]http://faculty.washington.edu/jstorey/edge/. Hypothesis testing on time-series expression of each gene was performed to test whether an average expression constitutes a flat line. The gene expression profile was fitted under a model based on null and alternative hypotheses. The null hypothesis states that there is no differential gene expression over a time period. The alternative hypothesis states that a gene is differentially expressed over a time period. The goodness of fit of 2 models was compared by F-statistic using a significant cut-off based on a false discovery rate criterion [82,83].

### The small-sample inference by graphical Gaussian model (GGM)

The R package “GeneNet” [33]—available at the R archive (CRAN) http://CRAN.R-project.org/—was used to construct the gene association network. The GeneNet was developed from the small-sample inference framework of graphical Gaussian model (GGM) to obtain a partial correlation coefficient, which is a correlation between 2 variables obtained when eliminating effects of other variables. In the case of 3 variables—x, y, and z—the partial correlation of x and y when eliminating the effect of z, *pr*_*xy,z*_ can be calculated as follows

(1) $$pr_{xy,z}=\frac{r_{\mathrm{xy}}-r_{\mathrm{xz}}r_{\mathrm{yz}}}{\sqrt{\left(1-r_{\mathrm{xz}}^{2})(1-r_{\mathrm{yz}}^{2}\right)}}$$

where *r* is the correlation coefficient between 2 variables. In the case of more than 3 variables, the partial correlation can be calculated from the following equation.

(2) $$pr_{xy,g}=-\frac{s_{\mathrm{xy}}}{\sqrt{s_{\mathrm{xx}}s_{\mathrm{yy}}}}$$

The *pr*_*xy,g*_ is the partial correlation between x and y against variable 3 to g. The *s*_*xy*_ = the xy^th^ element of the inverse of variance matrix (S = V^-1^). The element in matrix V is v_ij_ (i, j = 1, … , n) corresponding to a covariance between variables i and j.

For microarray, the number of variables (i.e. genes) is much higher than the number of measurements (i.e. microarray conditions), thus making the inversion step of matrix V invalid. In the new framework of GGM, the parameter estimation techniques were used to obtain partial correlation of small sample size. In order to decide which edges are significant to be included in the resulting GGM network, statistical significance was further assigned to the edges in the GGM network by fitting a mixture model (as shown below) to the observed partial correlation coefficients [33].

The distribution of observed partial correlation coefficients$\;f\left(\tilde{r}\right)$ is

(3) $$f\left(\tilde{r}\right)=\eta_{0}f_{0}\left(\tilde{r};\kappa \right)+\left(1-\eta_{0}\right)f_{A}(\tilde{r})$$

where $\tilde{r}$ is the observed partial correlation, $\eta_{0}$ is the unknown proportion of null edges, $f_{0}$ is the distribution under the null hypothesis of zero-partial correlation, *κ* is the degree of freedom, and $f_{A}$ is the distribution of observed partial correlations assigned to actually existing edges. The two-sided *p*-values for each edge corresponding to the null distribution $f_{0}$ were subsequently calculated and followed by false discovery rate multiple testing [82,83] to obtain *q*-values. The edges with *q*-values are equal or lower than 0.05 were presented in the resulting GGM network in this study.

### *Arabidopsis* lines and growth conditions

*Arabidopsis* ecotype Columbia-0 was used in this study. *Arabidopsis* mutant lines were obtained from the T-DNA or transposon inserted mutant collection (Col-0 background) of The Arabidopsis Biological Resource Centre (ABRC) [60] and The Nottingham Arabidopsis Stock Centre (NASC) [61]. Accession numbers of *Atidd5*, *c2h2*, *col*, *col7, wlim2a*, and *kh-ccch* are SALK_110990, SALK_070916, SALK_061956, SM_3_37788, SALK_067756, and SAIL_672_A10, respectively. Details of all mutant lines are shown in Additional file 8: Table S4. The seeds were vernalized in the dark for 3 days at 4°C before germination. Plants were grown on an equal mixture of sterile vermiculite and peat-based growing medium (PRO-MIX Bx/Microrise Pro, Premier) in a growth cabinet (SANYO) set at 60% humidity and 20-22°C with a light intensity of 100 μmol m^-2^ sec^-1^ and under 12 hr light/12 hr dark (short day) or 16 hr light/8 hr dark (long day) cycles. The trays of plant pots were sub-irrigated with a half-strength *Arabidopsis* liquid nutrient culture [84]. Leaves at a developmental stage of 3.90 [62] were harvested 4 times a day—1 hr before and after day break and night break. Leaves for starch analysis were harvested at the end of the light period.

### Expression analysis by quantitative RT-PCR

Total RNA was extracted from 100–200 mg leaf material (3 biological replicates) using Plant RNeasy kit (*Qiagen*), treated with DNaseI (*Invitrogen*), and reverse transcribed by Omniscript Reverse Transcriptase (*Qiagen*). Subsequently, real-time PCR was carried out using SYBR® Premix Ex Taq ™ II (Perfect Real Time) (*Takara*) using ubiquitin 2 (*UBQ2*) as a constitutive internal control. Details of the primer pairs used in qRT-PCR experiments are shown in Additional file 9: Table S5.

### Starch extraction and measurement

Starch from *Arabidopsis* leaves (3 biological replicates) was extracted using the method described by Smith and Zeeman [85]. Gelatinized starch was hydrolyzed to glucose by incubation for 4 hr at 37°C with α-amylase and α-amyloglucosidase. After the enzymatic digestion of starch to glucose, the amount of glucose was quantified by the capillary electrophoresis photodiode array detection (CE-DAD) system according to the manufacturer’s protocol (Agilent) [86]. Leaf starch content was calculated from the amount of glucose measured in this enzymatically-digested extract.

### Starch granule morphology analysis by Transmission Electron Microscope (TEM)

Fully expanded *Arabidopsis* leaves were collected at end of day, cut into 2 x 2 mm^2^ pieces, and immediately fixed with a cold solution of glutaraldehyde. Various parameters describing starch granule morphology (i.e. area, perimeter, width, height, and circularity) and number of starch granules per chloroplast were measured from TEM micrographs using ImageJ software (version 1.45). It was noted that circularity is calculated by the following formula:

(4) $$Circularity=\frac{4\pi \times area}{perimeter^{2}}$$

A value approaches 1.0 meaning a perfect circle and 0.0 meaning an elongated shape. The morphology data was tested for a statistically difference using a non-parametric Mann–Whitney U statistic (P-value < 0.05).

## Competing interests

The authors declare that they have no competing interests.

## Authors’ contributions

PI carried out the microarray data analysis, the model reconstruction, qRT-PCR experiments, and starch content and granule morphology analysis, and drafted the manuscript. PI, SC, and HT conceived and designed the research. SN and HT edited the manuscript. SP provided assistance in the statistical analysis of the research. JC provided assistance in R programming. HT provided assistance in growing *Arabidopsis*, qRT-PCR experiment, and starch measurement. AM, MT, and SB provided oversight of the work. All authors read and approved the final manuscript.

## Supplementary Material

Additional file 1 **Figure S1.** The gene association network of 11 carbon-related metabolisms inferred from GGM (Q < 0.05). Click here for file

Additional file 2 **Table S1.** Genes in the starch sub-network. Click here for file

Additional file 3 **Table S2.** Prediction of TF binding sites in 12 starch metabolic genes (2 kb-upstream). Click here for file

Additional file 4 **Figure S2.** Expression patterns of 2 TFs, At3g07650 (COL9) and At1g05805 (bHLH), and their target genes. Click here for file

Additional file 5 **Figure S3.** Expression patterns of starch genes in other regulatory modules. Click here for file

Additional file 6 **Figure S4.** Expression pattern of *C2H2* gene in the wild type, *Atidd5*, and *col* mutants quantified by qRT-PCR. Click here for file

Additional file 7 **Table S3.** Descriptive statistics of chloroplast morphology, starch granule morphology, and starch granule number in the wild type, *Atidd5*, and *col* mutants. Click here for file

Additional file 8 **Table S4.** T-DNA insertion lines of 6 candidate TFs that were utilized in this experiment. Click here for file

Additional file 9 **Table S5.** Primer pairs for quantitative RT-PCR. Click here for file

## References

[B1] Smith AM Kruger NJ, Hill SA, Ratcliffe RG Regulation of starch synthesis in storage organs Regulation of Primary Metabolic Pathways in Plants 1999 42 Kluwer Academic Publishers, Dordrecht 173 193 Proceedings of the Phytochemical Society of Europe]

[B2] Smith AM Denyer K Zeeman SC Edwads A Martin C The synthesis of the starch granule Plant Carbohydrate Biochemistry 1999 BIOS Scienctific Publishers Ltd, Oxford 79 89

[B3] Smith AM Zeeman SC Thorneycroft D Smith SM Starch mobilization in leaves J Exp Bot 2003 54 577 583 10.1093/jxb/erg036 12508068

[B4] Niittylä T Messerli G Trevisan M Chen J Smith AM Zeeman SC A previously unknown maltose transporter essential for starch degradation in leaves Science 2004 303 87 89 10.1126/science.1091811 14704427

[B5] Sokolov LN Dominguez-Solis JR Allary A-L Buchanan BB Luan S A redox-regulated chloroplast protein phosphatase binds to starch diurnally and functions in its accumulation Proc Natl Acad Sci USA 2006 103 9732 9737 10.1073/pnas.0603329103 16772378 PMC1480475

[B6] Roldán I Wattebled F Mercedes Lucas M Delvallé D Planchot V Jiménez S Pérez R Ball S D'Hulst C Mérida A The phenotype of soluble starch synthase IV defective mutants of Arabidopsis thaliana suggests a novel function of elongation enzymes in the control of starch granule formation Plant J 2007 49 492 504 10.1111/j.1365-313X.2006.02968.x 17217470

[B7] Fulton DC Stettler M Mettler T Vaughan CK Li J Francisco P Gil M Reinhold H Eicke S Messerli G β-AMYLASE4, a noncatalytic protein required for starch breakdown, acts upstream of three active β-amylases in Arabidopsis chloroplasts Plant Cell 2008 20 1040 1508 10.1105/tpc.107.056507 18390594 PMC2390740

[B8] Lohmeier-Vogel E Kerk D Nimick M Wrobel S Vickerman L Muench D Moorhead G Arabidopsis At5g39790 encodes a chloroplast-localized, carbohydrate-binding, coiled-coil domain-containing putative scaffold protein BMC Plant Biol 2008 8 120 10.1186/1471-2229-8-120 19038037 PMC2653042

[B9] Kötting O Santelia D Edner C Eicke S Marthaler T Gentry MS Comparot-Moss S Chen J Smith AM Steup M STARCH-EXCESS4 is a laforin-like phosphoglucan phosphatase required for starch degradation in Arabidopsis thaliana Plant Cell 2009 21 334 346 10.1105/tpc.108.064360 19141707 PMC2648081

[B10] Li L Foster CM Gan Q Nettleton D James MG Myers AM Wurtele ES Identification of the novel protein QQS as a component of the starch metabolic network in Arabidopsis leaves Plant J 2009 58 485 498 10.1111/j.1365-313X.2009.03793.x 19154206

[B11] Szydlowski N Ragel P Raynaud S Lucas MM Roldán I Montero M Muñoz FJ Ovecka M Bahaji A Planchot V Starch granule initiation in Arabidopsis requires the presence of either class IV or class III starch synthases Plant Cell 2009 21 2443 2457 10.1105/tpc.109.066522 19666739 PMC2751949

[B12] Tetlow IJ Morell MK Emes MJ Recent developments in understanding the regulation of starch metabolism in higher plants J Exp Bot 2004 55 2131 2145 10.1093/jxb/erh248 15361536

[B13] Geigenberger P Kolbe A Tiessen A Redox regulation of carbon storage and partitioning in response to light and sugars J Exp Bot 2005 56 1469 1479 10.1093/jxb/eri178 15863446

[B14] Kötting O Kossmann J Zeeman SC Lloyd JR Regulation of starch metabolism: the age of enlightenment? Curr Opin Plant Biol 2010 13 320 328 10.1016/j.pbi.2010.01.003 20171927

[B15] Ghosh HP Preiss J Adenosine diphosphate glucose pyrophosphorylase: a regulatory enzymein the biosynthesis of starch in spinach leaf chloroplasts J Biol Chem 1966 241 4491 4504 5922972

[B16] Fu Y Ballicora MA Leykam JF Preiss J Mechanism of reductive activation of potato tuber ADP-glucose pyrophosphorylase J Biol Chem 1998 273 25045 25052 10.1074/jbc.273.39.25045 9737961

[B17] Tiessen A Hendriks JHM Stitt M Branscheid A Gibon Y Farré EM Geigenberger P Starch synthesis in potato tubers is regulated by post-translational redox modification of ADP-glucose pyrophosphorylase: a novel regulatory mechanism linking starch synthesis to the sucrose supply Plant Cell 2002 14 2191 2213 10.1105/tpc.003640 12215515 PMC150765

[B18] Schindler I Renz A Schmid FX Beck E Activation of spinach pullulanase by reduction results in a decrease in the number of isomeric forms Biochim Biophys Acta Protein Struct Mol Enzymol 2001 1548 175 186 10.1016/S0167-4838(01)00228-X 11513962

[B19] Wu C Colleoni C Myers AM James MG Enzymatic properties and regulation of ZPU1, the maize pullulanase-type starch debranching enzyme Arch Biochem Biophys 2002 406 21 32 10.1016/S0003-9861(02)00412-5 12234486

[B20] Mikkelsen R Mutenda KE Mant A Schürmann P Blennow A α-Glucan, water dikinase (GWD): A plastidic enzyme with redox-regulated and coordinated catalytic activity and binding affinity Proc Natl Acad Sci USA 2005 102 1785 1790 10.1073/pnas.0406674102 15665090 PMC547843

[B21] Sparla F Costa A Lo Schiavo F Pupillo P Trost P Redox regulation of a novel plastid-targeted β-amylase of Arabidopsis Plant Physiol 2006 141 840 850 10.1104/pp.106.079186 16698902 PMC1489908

[B22] Sun C Palmqvist S Olsson H Borén M Ahlandsberg S Jansson C A novel WRKY transcription factor, SUSIBA2, participates in sugar signaling in barley by binding to the sugar-responsive elements of the iso1 promoter Plant Cell 2003 15 2076 2092 10.1105/tpc.014597 12953112 PMC181332

[B23] Zhu Y Cai X-L Wang Z-Y Hong M-M An Interaction between a MYC protein and an EREBP protein is involved in transcriptional regulation of the rice Wx gene J Biol Chem 2003 278 47803 47811 10.1074/jbc.M302806200 12947109

[B24] Tenorio G Orea A Romero JM Mérida Á Oscillation of mRNA level and activity of granule-bound starch synthase I in Arabidopsis leaves during the day/night cycle Plant Mol Biol 2003 51 949 958 10.1023/A:1023053420632 12777053

[B25] Ideker T Thorsson V Ranish JA Christmas R Buhler J Eng JK Bumgarner R Goodlett DR Aebersold R Hood L Integrated genomic and proteomic analyses of a systematically perturbed metabolic network Science 2001 292 929 934 10.1126/science.292.5518.929 11340206

[B26] Lee TI Rinaldi NJ Robert F Odom DT Bar-Joseph Z Gerber GK Hannett NM Harbison CT Thompson CM Simon I Transcriptional regulatory networks in Saccharomyces cerevisiae Science 2002 298 799 804 10.1126/science.1075090 12399584

[B27] Gardner TS di Bernardo D Lorenz D Collins JJ Inferring genetic networks and identifying compound mode of action via expression profiling Science 2003 301 102 105 10.1126/science.1081900 12843395

[B28] Wang Y Joshi T Zhang X-S Xu D Chen L Inferring gene regulatory networks from multiple microarray datasets Bioinformatics 2006 22 2413 2420 10.1093/bioinformatics/btl396 16864593

[B29] Ma S Gong Q Bohnert HJ An Arabidopsis gene network based on the graphical Gaussian model Genome Res 2007 17 1614 1625 10.1101/gr.6911207 17921353 PMC2045144

[B30] Carrera J Rodrigo G Jaramillo A Elena S Reverse-engineering the Arabidopsis thaliana transcriptional network under changing environmental conditions Genome Biol 2009 10 R96 10.1186/gb-2009-10-9-r96 19754933 PMC2768985

[B31] Needham C Manfield I Bulpitt A Gilmartin P Westhead D From gene expression to gene regulatory networks in Arabidopsis thaliana BMC Syst Biol 2009 3 85 10.1186/1752-0509-3-85 19728870 PMC2760521

[B32] Mao L Van Hemert J Dash S Dickerson J Arabidopsis gene co-expression network and its functional modules BMC Bioinformatics 2009 10 346 10.1186/1471-2105-10-346 19845953 PMC2772859

[B33] Schafer J Strimmer K An empirical Bayes approach to inferring large-scale gene association networks Bioinformatics 2005 21 754 764 10.1093/bioinformatics/bti062 15479708

[B34] Opgen-Rhein R Strimmer K Infering gene dependency networks from genomic longitudinal data: a functional data approach REVSTAT 2006 4 53 65

[B35] Zeeman SC Tiessen A Pilling E Kato KL Donald AM Smith AM Starch synthesis in Arabidopsis. granule synthesis, composition, and structure Plant Physiol 2002 129 516 529 10.1104/pp.003756 12068097 PMC161669

[B36] Smith SM Fulton DC Chia T Thorneycroft D Chapple A Dunstan H Hylton C Zeeman SC Smith AM Diurnal changes in the transcriptome encoding enzymes of starch metabolism provide evidence for both transcriptional and posttranscriptional regulation of starch metabolism in Arabidopsis leaves Plant Physiol 2004 136 2687 2699 10.1104/pp.104.044347 15347792 PMC523333

[B37] Blasing OE Gibon Y Gunther M Hohne M Morcuende R Osuna D Thimm O Usadel B Scheible W-R Stitt M Sugars and circadian regulation make major contributions to the global regulation of diurnal gene expression in Arabidopsis Plant Cell 2005 17 3257 3281 10.1105/tpc.105.035261 16299223 PMC1315368

[B38] Gibon Y Bläsing OE Palacios -Rojas N Pankovic D Hendriks JHM Fisahn J Höhne M Günther M Stitt M Adjustment of diurnal starch turnover to short days: depletion of sugar during the night leads to a temporary inhibition of carbohydrate utilization, accumulation of sugars and post-translational activation of ADP-glucose pyrophosphorylase in the following light period Plant J 2004 39 847 862 10.1111/j.1365-313X.2004.02173.x 15341628

[B39] Storey JD Xiao W Leek JT Tompkins RG Davis RW Significance analysis of time course microarray experiments Proc Natl Acad Sci USA 2005 102 12837 12842 10.1073/pnas.0504609102 16141318 PMC1201697

[B40] Leek JT Monsen E Dabney AR Storey JD EDGE: extraction and analysis of differential gene expression Bioinformatics 2006 22 507 508 10.1093/bioinformatics/btk005 16357033

[B41] Thimm O Blasing O Gibon Y Nagel A Meyer S Kruger P Selbig J Muller LA Rhee SY Stitt M Mapman: a user-driven tool to display genomics data sets onto diagrams of metabolic pathways and other biological processes Plant J 2004 37 914 939 10.1111/j.1365-313X.2004.02016.x 14996223

[B42] Lao NT Schoneveld O Mould RM Hibberd JM Gray JC Kavanagh TA An Arabidopsis gene encoding a chloroplast-targeted β-amylase Plant J 1999 20 519 527 10.1046/j.1365-313X.1999.00625.x 10652124

[B43] Kossmann J Lloyd J Understanding and influencing starch biochemistry Crit Rev Biochem Mol Biol 2000 35 141 196 10907795

[B44] Lu Y Gehan JP Sharkey TD Daylength and circadian effects on starch degradation and maltose metabolism Plant Physiol 2005 138 2280 2291 10.1104/pp.105.061903 16055686 PMC1183414

[B45] Scheidig A Fröhlich A Schulze S Lloyd JR Kossmann J Downregulation of a chloroplast-targeted beta-amylase leads to a starch-excess phenotype in leaves Plant J 2002 30 581 591 10.1046/j.1365-313X.2002.01317.x 12047632

[B46] Kaplan F Sung DY Guy CL Roles of beta-amylase and starch breakdown during temperatures stress Physiol Plantarum 2006 126 120 128 10.1111/j.1399-3054.2006.00604.x

[B47] Makino S Kiba T Imamura A Hanaki N Nakamura A Suzuki T Taniguchi M Ueguchi C Sugiyama T Mizuno T Genes encoding pseudo-response regulators: insight into His-to-Asp phosphorelay and circadian rhythm in Arabidopsis thaliana Plant Cell Physiol 2000 41 791 803 10.1093/pcp/41.6.791 10945350

[B48] Strayer C Oyama T Schultz TF Raman R Somers DE Más P Panda S Kreps JA Kay SA Cloning of the Arabidopsis clock gene TOC1, an autoregulatory response regulator homolog Science 2000 289 768 771 10.1126/science.289.5480.768 10926537

[B49] Ito S Kawamura H Niwa Y Nakamichi N Yamashino T Mizuno T A genetic study of the Arabidopsis circadian clock with reference to the TIMING OF CAB EXPRESSION 1 (TOC1) gene Plant Cell Physiol 2009 50 290 303 19098071 10.1093/pcp/pcn198

[B50] Harmer SL Hogenesch JB Straume M Chang H-S Han B Zhu T Wang X Kreps JA Kay SA Orchestrated transcription of key pathways in Arabidopsis by the circadian clock Science 2000 290 2110 2113 10.1126/science.290.5499.2110 11118138

[B51] Kaplan F Guy CL RNA interference of Arabidopsis beta-amylase8 prevents maltose accumulation upon cold shock and increases sensitivity of PSII photochemical efficiency to freezing stress Plant J 2005 44 730 743 10.1111/j.1365-313X.2005.02565.x 16297066

[B52] Papuga J Hoffmann C Dieterle M Moes D Moreau F Tholl S Steinmetz A Thomas C Arabidopsis LIM proteins: a family of actin bundlers with distinct expression patterns and modes of regulation Plant Cell 2010 22 3034 3052 10.1105/tpc.110.075960 20817848 PMC2965535

[B53] Dean Rider S Henderson JT Jerome RE Edenberg HJ Romero-Severson J Ogas J Coordinate repression of regulators of embryonic identity by PICKLE during germination in Arabidopsis Plant J 2003 35 33 43 10.1046/j.1365-313X.2003.01783.x 12834400 PMC2515612

[B54] Steffens NO Galuschka C Schindler M Bulow L Hehl R AthaMap: an online resource for in silico transcription factor binding sites in the Arabidopsis thaliana genome Nucl Acids Res 2004 32 D368 372 10.1093/nar/gkh017 14681436 PMC308752

[B55] Steffens NO Galuschka C Schindler M Bulow L Hehl R AthaMap web tools for database-assisted identification of combinatorial cis-regulatory elements and the display of highly conserved transcription factor binding sites in Arabidopsis thaliana Nucl Acids Res 2005 33 W397 402 10.1093/nar/gki395 15980498 PMC1160156

[B56] Bülow L Steffens NO Galuschka C Schindler M Hehl R AthaMap: from in silico data to real transcription factor binding sites In Silico Biol 2006 6 243 252 10.1007/3-540-28185-1_10 16922688

[B57] Galuschka C Schindler M Bulow L Hehl R AthaMap web tools for the analysis and identification of co-regulated genes Nucl Acids Res 2007 35 D857 862 10.1093/nar/gkl1006 17148485 PMC1761422

[B58] Obayashi T Hayashi S Saeki M Ohta H Kinoshita K ATTED-II provides coexpressed gene networks for Arabidopsis Nucl Acids Res 2009 37 D987 D991 10.1093/nar/gkn807 18953027 PMC2686564

[B59] Obayashi T Kinoshita K Rank of correlation coefficient as a comparable measure for biological significance of gene coexpression DNA Res 2009 16 249 260 10.1093/dnares/dsp016 19767600 PMC2762411

[B60] Alonso JM Stepanova AN Leisse TJ Kim CJ Chen H Shinn P Stevenson DK Zimmerman J Barajas P Cheuk R Genome-wide insertional mutagenesis of Arabidopsis thaliana Science 2003 301 653 657 10.1126/science.1086391 12893945

[B61] Tissier AF Marillonnet S Klimyuk V Patel K Torres MA Murphy G Jones JDG Multiple independent defective suppressor-mutator transposon insertions in Arabidopsis: a tool for functional genomics Plant Cell 1999 11 1841 1852 10521516 10.1105/tpc.11.10.1841 PMC144107

[B62] Boyes DC Zayed AM Ascenzi R McCaskill AJ Hoffman NE Davis KR Görlach J Growth stage-based phenotypic analysis of Arabidopsis: a model for high throughput functional genomics in plants Plant Cell 2001 13 1499 1510 11449047 10.1105/TPC.010011 PMC139543

[B63] Englbrecht C Schoof H Bohm S Conservation, diversification and expansion of C2H2 zinc finger proteins in the Arabidopsis thaliana genome BMC Genomics 2004 5 39 10.1186/1471-2164-5-39 15236668 PMC481060

[B64] Colasanti J Tremblay R Wong A Coneva V Kozaki A Mable B The maize INDETERMINATE1 flowering time regulator defines a highly conserved zinc finger protein family in higher plants BMC Genomics 2006 7 158 10.1186/1471-2164-7-158 16784536 PMC1586020

[B65] Welch D Hassan H Blilou I Immink R Heidstra R Scheres B Arabidopsis JACKDAW and MAGPIE zinc finger proteins delimit asymmetric cell division and stabilize tissue boundaries by restricting SHORT-ROOT action Genes Dev 2007 21 2196 2204 10.1101/gad.440307 17785527 PMC1950858

[B66] Levesque MP Vernoux T Busch W Cui H Wang JY Blilou I Hassan H Nakajima K Matsumoto N Lohmann JU Whole-genome analysis of the SHORT-ROOT developmental pathway in Arabidopsis PLoS Biol 2006 4 e143 10.1371/journal.pbio.0040143 16640459 PMC1450008

[B67] Tanimoto M Tremblay R Colasanti J Altered gravitropic response, amyloplast sedimentation and circumnutation in the Arabidopsis shoot gravitropism 5 mutant are associated with reduced starch levels Plant Mol Biol 2008 67 57 69 10.1007/s11103-008-9301-0 18259878

[B68] Seo PJ Kim MJ Ryu J-Y Jeong E-Y Park C-M Two splice variants of the IDD14 transcription factor competitively form nonfunctional heterodimers which may regulate starch metabolism Nat Commun 2011 2 303 21556057 10.1038/ncomms1303

[B69] Seo PJ Ryu J Kang SK Park C-M Modulation of sugar metabolism by an INDETERMINATE DOMAIN transcription factor contributes to photoperiodic flowering in Arabidopsis Plant J 2011 65 418 429 10.1111/j.1365-313X.2010.04432.x 21265895

[B70] Kakizaki T Matsumura H Nakayama K Che F-S Terauchi R Inaba T Coordination of plastid protein import and nuclear gene expression by plastid-to-nucleus retrograde signaling Plant Physiol 2009 151 1339 1353 10.1104/pp.109.145987 19726569 PMC2773054

[B71] Zentella R Zhang Z-L Park M Thomas SG Endo A Murase K Fleet CM Jikumaru Y Nambara E Kamiya Y Sun T-P Global analysis of DELLA direct targets in early gibberellin signaling in Arabidopsis Plant Cell 2007 19 3037 3057 10.1105/tpc.107.054999 17933900 PMC2174696

[B72] Hruz T Laule O Szabo G Wessendorp F Bleuler S Oertle L Widmayer P Gruissem W Zimmermann P Genevestigator V3: a reference expression database for the meta-analysis of transcriptome Adv Bioinformatics 2008 5 10.1155/2008/420747 PMC2777001 19956698

[B73] Smyth DR Bowman JL Meyerowitz EM Early flower development in Arabidopsis Plant Cell 1990 2 755 767 2152125 10.1105/tpc.2.8.755 PMC159928

[B74] Nakayama N Arroyo JM Simorowski J May B Martienssen R Irish VF Gene trap lines define domains of gene regulation in Arabidopsis petals and stamens Plant Cell 2005 17 2486 2506 10.1105/tpc.105.033985 16055634 PMC1197429

[B75] Jaspers P Blomster T Brosché M Salojärvi J Ahlfors R Vainonen JP Reddy RA Immink R Angenent G Turck F Unequally redundant RCD1 and SRO1 mediate stress and developmental responses and interact with transcription factors Plant J 2009 60 268 279 10.1111/j.1365-313X.2009.03951.x 19548978

[B76] Belles-Boix E Babiychuk E Van Montagu M Inzé D Kushnir S CEO1, a new protein from Arabidopsis thaliana, protects yeast against oxidative damage FEBS Lett 2000 482 19 24 10.1016/S0014-5793(00)02016-0 11018516

[B77] Overmyer K Tuominen H Kettunen R Betz C Langebartels C Sandermann H Kangasjarvi J Ozone-sensitive Arabidopsis rcd1 mutant reveals opposite roles for ethylene and jasmonate signaling pathways in regulating superoxide-dependent cell death Plant Cell 2000 12 1849 1862 11041881 10.1105/tpc.12.10.1849 PMC149124

[B78] Ahlfors R Lang S Overmyer K Jaspers P Brosche M Tauriainen A Kollist H Tuominen H Belles-Boix E Piippo M Arabidopsis RADICAL-INDUCED CELL DEATH1 belongs to the WWE protein–protein interaction domain protein family and modulates abscisic acid, ethylene, and methyl jasmonate responses Plant Cell 2004 16 1925 1937 10.1105/tpc.021832 15208394 PMC514171

[B79] Yilmaz A Mejia-Guerra MK Kurz K Liang X Welch L Grotewold E AGRIS: the Arabidopsis gene regulatory information server, an update Nucl Acids Res 2011 39 D1118 D1122 10.1093/nar/gkq1120 21059685 PMC3013708

[B80] Workman C Jensen LJ Jarmer H Berka R Gautier L Nielser HB Saxild H-H Nielsen C Brunak S Knudsen S A new non-linear normalization method for reducing variability in DNA microarray experiments Genome Biol 2002 3 research0048.0041 research0048.0016 12225587 10.1186/gb-2002-3-9-research0048 PMC126873

[B81] Li C Wong WH Model-based analysis of oligonucleotide arrays: expression index computation and outlier detection Proc Natl Acad Sci USA 2001 98 31 36 10.1073/pnas.98.1.31 11134512 PMC14539

[B82] Benjamini Y Hochberg Y Controlling the false discovery rate: a practical and powerful approach to multiple testing J Royal Stat Soc Ser B 1995 57 289 300

[B83] Storey JD A direct approach to false discovery rates J Royal Stat Soc Ser B 2002 64 479 498 10.1111/1467-9868.00346

[B84] Fujiwara T Hirai MY Chino M Komeda Y Naito S Effects of sulfur nutrition on expression of the soybean seed storage protein genes in transgenic petunia Plant Physiol 1992 99 263 268 10.1104/pp.99.1.263 16668860 PMC1080434

[B85] Smith AM Zeeman SC Quantification of starch in plant tissues Nat Protoc 2006 1 1342 1345 10.1038/nprot.2006.232 17406420

[B86] Sato S Soga T Nishioka T Tomita M Simultaneous determination of the main metabolites in rice leaves using capillary electrophoresis mass spectrometry and capillary electrophoresis diode array detection Plant J 2004 40 151 163 10.1111/j.1365-313X.2004.02187.x 15361149

