# Supplementary material for: Inferring transcriptional gene regulation network of starch metabolism in Arabidopsis thaliana leaves using graphical Gaussian model
Source: BMC Syst Biol. 2012 Aug 16;6:100. doi: 10.1186/1752-0509-6-100 (PMC3490714; doi:10.1186/1752-0509-6-100)
Supplement: Additional file 1 — Figure S1. The gene association network of 11 carbon-related metabolisms inferred from GGM (Q < 0.05). [file 1752-0509-6-100-S1.pdf]

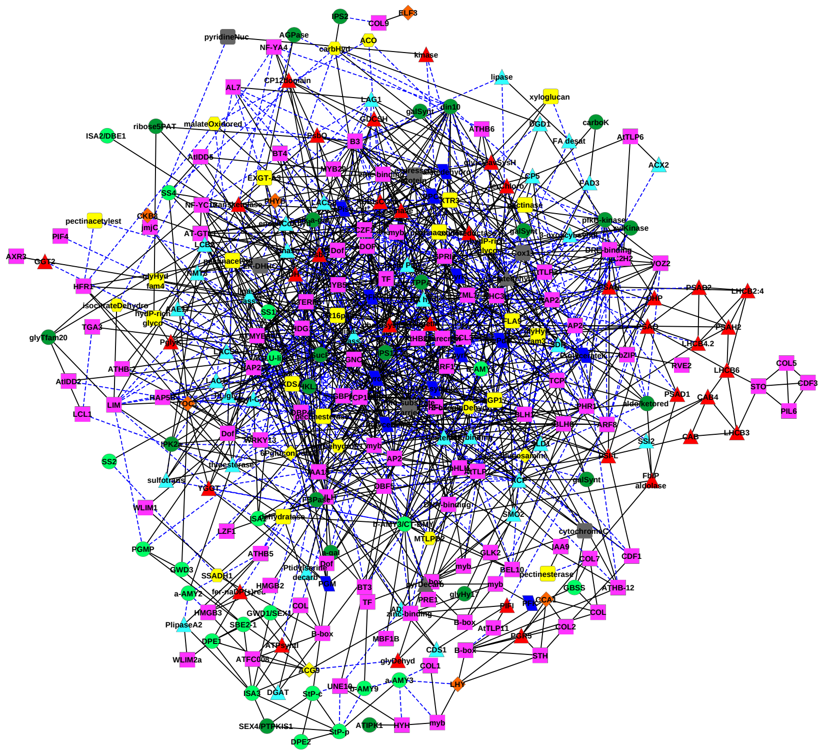

Supplementary figure 1.1. Gene association network of 106 TF, 6 clock, and 173 metabolic genes. The network contains 277 nodes and 1,174 edges. The metabolic genes were selected from 11 functional categories (defined by MapMan and shown in Table 2 in the main article). These 11 functional groups are related to carbon metabolism in a broader view.

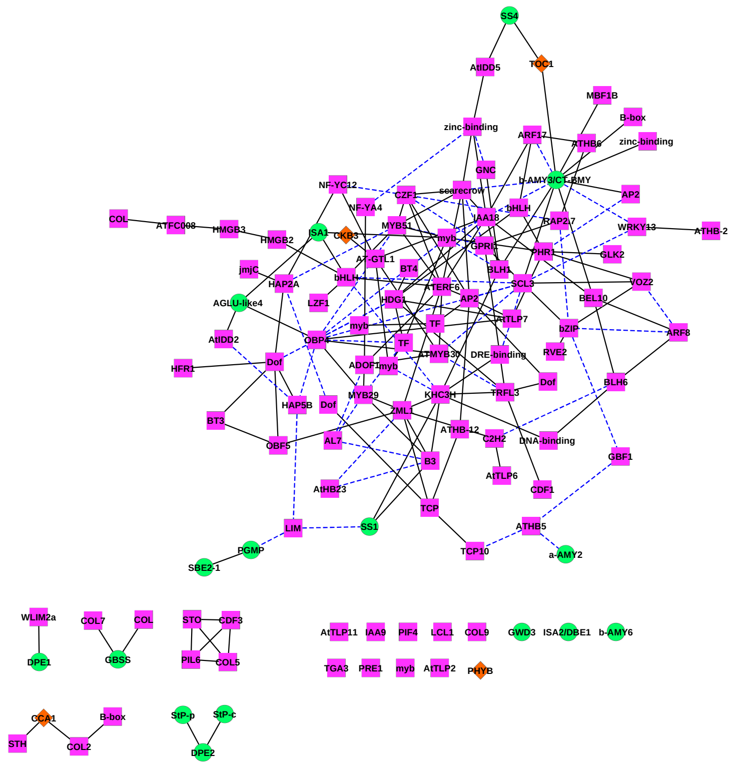

Supplementary figure 1.2. The intersection result of the networks constructed by starch genes (Figure 2 in the main article) and carbon-related genes (Supp. figure 1.1). This intersected network contains 177 nodes and 181 edges. All nodes in the starch network are in the carbon network and 68% of edges in the starch network were detected in the carbon network.

Node symbols

Clock	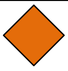
OPP	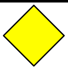
PS	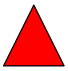
Starch	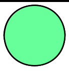
TCA	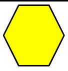
TF	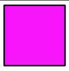
Cell wall	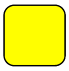
Fermentation	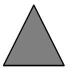
Gluconeogenesis	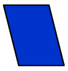
Glycolysis	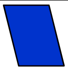
Lipid	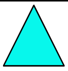
Major CHO	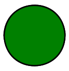
Minor CHO	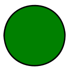
Mitochrondrial electron transport	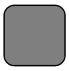
