# Supplementary figures and images for: Inferring transcriptional gene regulation network of starch metabolism in Arabidopsis thaliana leaves using graphical Gaussian model
Source: BMC Syst Biol. 2012 Aug 16;6:100. doi: 10.1186/1752-0509-6-100 (PMC3490714; doi:10.1186/1752-0509-6-100)

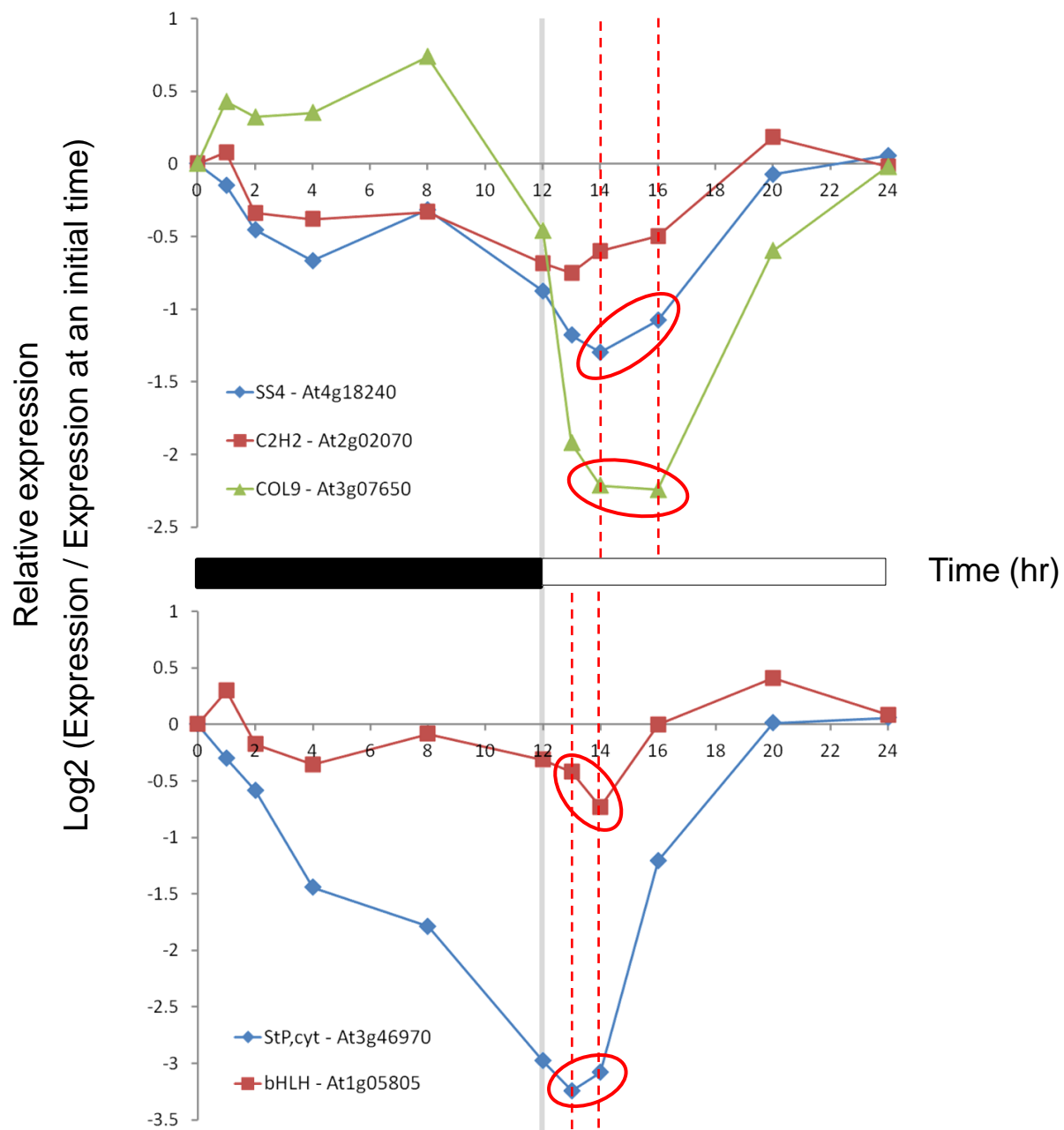

Supplement: Additional file 4 — Figure S2. Expression patterns of 2 TFs, At3g07650 (COL9) and At1g05805 (bHLH), and their target genes. [file 1752-0509-6-100-S4.pdf]

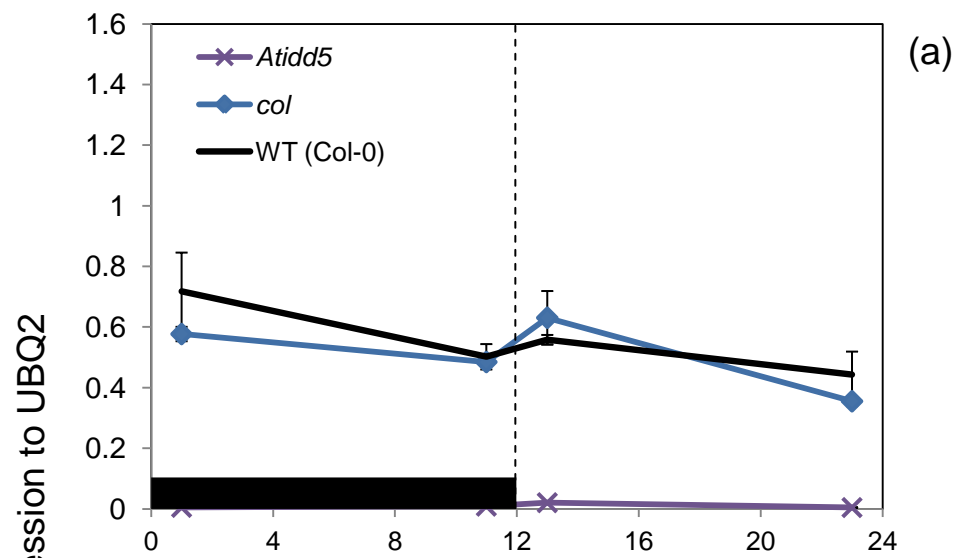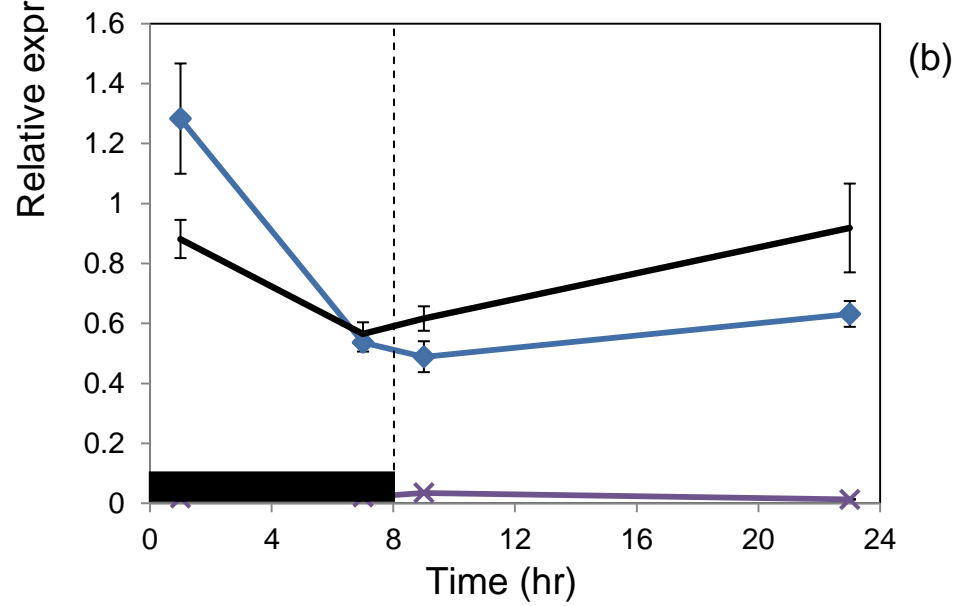

Supplement: Additional file 6 — Figure S4. Expression pattern of C2H2 gene in the wild type, Atidd5, and col mutants quantified by qRT-PCR. [file 1752-0509-6-100-S6.pdf]
