# Supplementary material for: Inferring transcriptional gene regulation network of starch metabolism in Arabidopsis thaliana leaves using graphical Gaussian model
Source: BMC Syst Biol. 2012 Aug 16;6:100. doi: 10.1186/1752-0509-6-100 (PMC3490714; doi:10.1186/1752-0509-6-100)
Supplement: Additional file 5 — Figure S3. Expression patterns of starch genes in other regulatory modules. [file 1752-0509-6-100-S5.pdf]

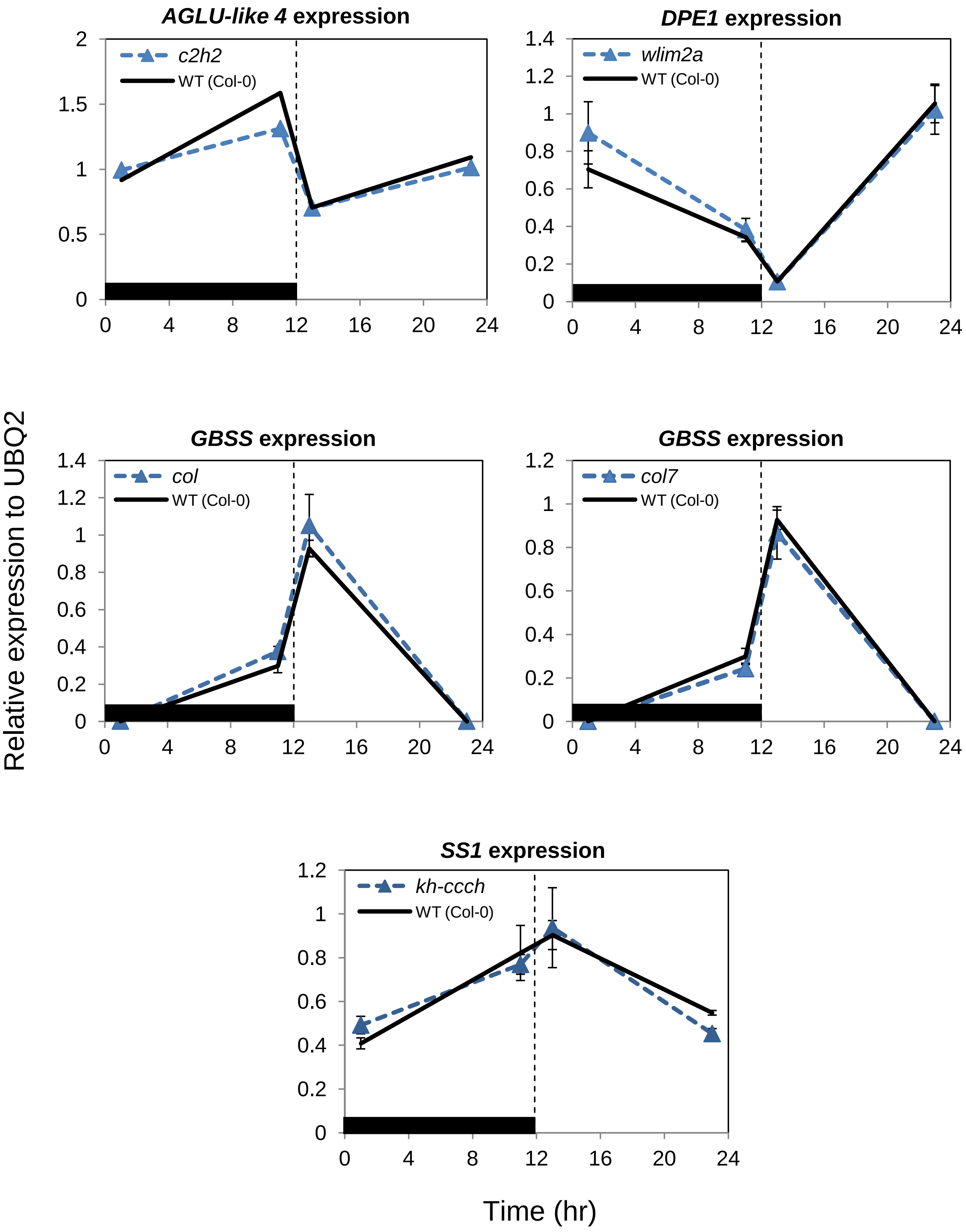

Supplementary figure 3. Gene expression patterns of *AGLU-like4*, *GBSS*, *DPE1*, and *SS1* in the wild type (black solid line) and *c2h2*, *col* & *col7*, *wlim2a*, and *kh-ccch* mutants (blue broken line with triangle), respectively.
