# Supplementary material for: Inferring transcriptional gene regulation network of starch metabolism in Arabidopsis thaliana leaves using graphical Gaussian model
Source: BMC Syst Biol. 2012 Aug 16;6:100. doi: 10.1186/1752-0509-6-100 (PMC3490714; doi:10.1186/1752-0509-6-100)
Supplement: Additional file 8 — Table S4. T-DNA insertion lines of 6 candidate TFs that were utilized in this experiment. [file 1752-0509-6-100-S8.doc]

**Supplementary table** **4**. T-DNA insertion transgenic lines of 6 candidate TFs that were utilized in this experiment

| **Candidate TFs** | | **T-DNA line** | **T-DNA Insert location** | **Source**^1^ | **Starch metabolic gene** | |
| --- | --- | --- | --- | --- | --- | --- |
| At2g02070 | AtIDD5 | SALK_110990 | Exon | SALK | At4g18240 | SS4 |
| At2g39900 | WLIM2a | SALK_067756 | Intron | SALK | At5g64860 | DPE1 |
| At2g21320 | COL | SALK_061956 | 300-UTR3 | SALK | At1g32900 | GBSS |
| At1g73870 | COL7 | SM_3_37788 | Exon | JIC | At1g32900 | GBSS |
| At5g06770 | KH-CCCH | SAIL_672_A10 | Intron | SAIL | At5g24300 | SS1 |
| At3g50700 | C2H2 | SALK_070916 | Exon | SALK | At5g11720 | AGLU-like4 |

1. Source of mutant seed ordered: SALK (Salk Institute Genomic Analysis Laboratory) and SAIL (the Syngenta Arabidopsis Insertion Library, US) = ordered from The Arabidopsis Biological Resource Center (ABRC), US; JIC (the John Innes Centre, UK) = ordered from The Nottingham Arabidopsis Stock Centre (NASC), UK.
