# Supplementary material for: Inferring transcriptional gene regulation network of starch metabolism in Arabidopsis thaliana leaves using graphical Gaussian model
Source: BMC Syst Biol. 2012 Aug 16;6:100. doi: 10.1186/1752-0509-6-100 (PMC3490714; doi:10.1186/1752-0509-6-100)
Supplement: Additional file 9 — Table S5. Primer pairs for quantitative RT-PCR. [file 1752-0509-6-100-S9.doc]

**Supplementary table 5**. Primer pairs for quantitative RT-PCR

| **AGI** | **Gene** | **Primer name** | **Primer Sequence** |
| --- | --- | --- | --- |
| At2g21320 | COL | col.9.2_qF | GGGAAGAGAACACATAGGCG |
|  |  | col.9.2_qR | GGAAGCCTTTTGACACCGTA |
| At1g73870 | COL7 | col7.10.3_qF | AACGCTGAGCAGAGACCTCG |
|  |  | col7.10.3_qR | CCATCGGCCCCAAGAGTTTCA |
| At5g06770 | KH-CCCH | kh.12.1_qF | TGCCTCTCACAACTCCAATG |
|  |  | kh.12.1_qR | TGGACATCCAGAAGTGCTGA |
| At2g02070 | AtIDD5 | c2h2.1.1_qF | GACCTCACCGGAATCAAGAA |
|  |  | c2h2.1.1_qR | GTGATGAAACTGTCTCGCCG |
| At3g50700 | C2H2 | c2h2.19.1_qR | TACTTGGAGAAGCCGAGGAA |
|  |  | c2h2.19.1_qF | GCTCCGGTGGATACTGAGTC |
| At2g39900 | WLIM2a | lim.6.1_qR | TGGAGATATCGGACAGCCTC |
|  |  | lim.6.1_qF | GACAAATGCGCTACTTGCAC |
| At1g32900 | GBSS | gb.1_qF | AGGCACCACAGGTTCTGAAC |
|  |  | gb.1_qR | TGTAGACTCCGCGGGATTGATA |
| at5g24300 | SS1 | ss1.2_qF | GCATGCCGGCCTTGTTCCCA |
|  |  | ss1.2_qR | TGCTGGCTCCACCCCCTGAT |
| At4g18240 | SS4 | ss4.1_qF | CGTGACTTAAGGGCTTTGGA |
|  |  | ss4.1_qR | GCAGCTCGGCTAAAATACGA |
| At5g11720 | AGLU-like4 | AGL4.1_qF | TCTCCACAACACGACGCCGTTT |
|  |  | AGL4.1_qR | GGTAACGCGGATGATAGTTGAAGG |
| At5g64860 | DPE1 | DPE1.1_qF | GTTTCGTGGTCCTCATGGCAT |
|  |  | DPE1.1_qR | GCTCATCCTTGATTAACAAGCCG |
| At2g36170 | UBQ2 | UBQ2_144_F | CCAAGATCCAGGACAAAGAAGGA |
|  |  | UBQ2_372_R | TGGAGACGAGCATAACACTTGC |
